# Low‐Crystalline AuCuIn Catalyst for Gaseous CO_2_ Electrolyzer

**DOI:** 10.1002/advs.202104908

**Published:** 2022-01-22

**Authors:** Gyeong Ho Han, Junhyeong Kim, Seohyeon Jang, Hyunki Kim, Wenwu Guo, Seokjin Hong, Junhyeop Shin, Inho Nam, Ho Won Jang, Soo Young Kim, Sang Hyun Ahn

**Affiliations:** ^1^ School of Chemical Engineering and Material Science Chung‐Ang University Seoul 06974 Republic of Korea; ^2^ Department of Intelligent Energy and Industry Chung‐Ang University Seoul 06974 Republic of Korea; ^3^ Department of Materials Science and Engineering Research Institute of Advanced Materials Seoul National University Seoul 08826 Republic of Korea; ^4^ Department of Materials Science and Engineering Korea University Seoul 02841 Republic of Korea

**Keywords:** electrochemical carbon dioxide reduction, electrodeposition, gas diffusion electrodes, low‐crystalline trimetallic catalysts, membrane electrode assembly‐based electrolyzers

## Abstract

Despite its importance for the establishment of a carbon‐neutral society, the electrochemical reduction of CO_2_ to value‐added products has not been commercialized yet because of its sluggish kinetics and low selectivity. The present work reports the fabrication of a low‐crystalline trimetallic (AuCuIn) CO_2_ electroreduction catalyst and demonstrates its high performance in a gaseous CO_2_ electrolyzer. The high Faradaic efficiency (FE) of CO formation observed at a low overpotential in a half‐cell test is ascribed to the controlled crystallinity and composition of this catalyst as well as to its faster charge transfer, downshifted d‐band center, and low oxophilicity. The gaseous CO_2_ electrolyzer with the optimal catalyst as the cathode exhibits superior cell performance with a high CO FE and production rate, outperforming state‐of‐the‐art analogs. Thus, the obtained results pave the way to the commercialization of CO_2_ electrolyzers and promote the establishment of a greener society.

## Graphical abstract

Low‐crystalline AuCuIn is proposed as effective catalyst for cathode in gaseous CO_2_ electrolyzer to produce CO selectively, owing to its suitable binding energy, fast electron transfer, enhanced mass transfer, and suppressed H_2_ evolution. The electrolyzer demonstrates that a CO partial current density reaches 220.1 mA cm^−2^ with a Faradaic efficiency of ≈100% at a low cell voltage of 2.8 V_cell_.

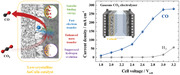

## 1 Introduction

The increase in atmospheric CO_2_ levels over the past century induced by the extensive use of fossil fuels has triggered global climate change, the mitigation of which is a task of high importance for securing a sustainable future.^[^ ^1^, ^2^, ^3^ ^]^ Consequently, the development of carbon capture, utilization, and storage technologies for reducing CO_2_ concentrations has drawn much attention.^[^ ^4^, ^5^, ^6^, ^7^ ^]^ Among these technologies, the electrochemical reduction of CO_2_ to useful products (e.g., CO, HCOOH, and C_2_H_4_) allows the efficient storage of electricity generated from intermittent renewable energy sources.^[^ ^6^, ^7^ ^]^ This method can be conducted at room temperature and atmospheric pressure, which offers the benefits of compact design and upscalability.^[^ ^8^ ^]^ However, the electrochemical reduction of CO_2_ still remains challenging because of the high stability of this molecule, leading to its high reduction overpotential.^[^ ^9^ ^]^ Moreover, the diversity of reduction products due to the complexity of the reaction mechanism results in selectivity problems.^[^ ^10^, ^11^ ^]^ In view of the above, much effort has been directed at the development of high‐performance (high‐activity, high‐selectivity, and low‐cost) catalysts for electrochemical CO_2_ reduction.

Among the reduction products, the market price and annual global production volumes^[^ ^12^ ^]^ of CO indicate that it is a valuable product with numerous industrial applications.^[^ ^13^, ^14^ ^]^ The selectivity of CO_2_ reduction mainly depends on the intrinsic properties of the chosen catalyst.^[^ ^15^ ^]^ For example, Au catalysts for CO_2_‐to‐CO reduction^[^ ^16^, ^17^ ^]^ have been extensively investigated because of their favorable binding energy of *COOH intermediate.^[^ ^11^ ^]^ Their catalytic properties can be tuned via particle size,^[^ ^18^, ^19^ ^]^ morphology,^[^ ^20^, ^21^, ^22^, ^23^ ^]^ oxidation state,^[^ ^21^, ^24^ ^]^ grain boundary,^[^ ^25^, ^26^ ^]^ and facet^[^ ^27^, ^28^ ^]^ control. However, the high cost of pure Au catalysts hinders their widespread application and has inspired the development of Au‐based bimetallic catalysts (AuCu,^[^ ^29^, ^30^, ^31^, ^32^, ^33^ ^]^ AuFe,^[^ ^34^ ^]^ AuPd,^[^ ^35^, ^36^ ^]^ AuMo,^[^ ^37^ ^]^ and AuCd^[^ ^38^ ^]^), the composition of which can be controlled to reduce the cost and modulate the electronic structure of the catalytic surface for further performance enhancement. AuCu systems afford the best Au‐based bimetallic catalysts.^[^ ^29^, ^30^, ^31^, ^32^, ^33^ ^]^ In particular, when the Au:Cu ratio approaches 3.0, the d‐band center is downshifted from the Fermi level, which results in optimal energies of reaction intermediate binding and hence, in maximal catalytic performance.^[^ ^29^, ^30^ ^]^ The synergetic effects of bimetallic catalyst components have inspired the exploration of trimetallic catalysts, despite the limited related examples.

Beyond catalyst development, recent reports have emphasized the rational design of CO_2_ electrolyzers and demonstrated that practical CO_2_ electrolyzers needs to produce the desired products at current densities of >200 mA cm^−2^.^[^ ^39^, ^40^, ^41^ ^]^ Particularly, to achieve CO production costs of <0.6 $ kg^−1^, a product selectivity of ≈90% needs to be maintained at operating voltages of <3.0 V.^[^ ^42^ ^]^ However, in conventional electrolyzers using CO_2_ dissolved in aqueous electrolytes, the low solubility of CO_2_ limits its diffusion rate and hence, the cathodic reaction rate, resulting in mass transfer issues. This leads to an insufficiently high maximum current density of ≈35 mA cm^−2^.^[^ ^39^, ^43^ ^]^ Therefore, to enhance the mass transfer of reactants, recent research has focused on the development of electrolyzers directly using gaseous CO_2_.^[^ ^44^ ^]^ In a typical cathode part configuration, the gas diffusion electrode (GDE) is located between the bulk catholyte and gaseous CO_2_ supply channel, which enables much faster reactant diffusion.^[^ ^45^, ^46^ ^]^ Although the gaseous CO_2_ electrolyzers feature high current densities for the desired products,^[^ ^45^, ^46^ ^]^ the presence of the bulk catholyte induces a large Ohmic drop, specifically in the high‐current‐density region. Consequently, membrane electrode assembly (MEA)‐based electrolyzers using humidified gaseous CO_2_ have received much attention owing to their advanced design.^[^ ^47^ ^]^ The Ohmic resistance can be minimized without using bulk electrolytes by employing the MEA, with a zero‐gap structure sandwiching the membrane between the cathode and anode.^[^ ^48^, ^49^ ^]^ In addition, the accelerated formation of a three‐phase boundary (TPB) at the interface between the catalyst layer and humidified CO_2_ generates abundant active sites to promote reaction kinetics.^[^ ^39^, ^50^ ^]^

Herein, we describe a trimetallic catalyst for electrochemical CO_2_ reduction and probe its performance as the cathode of an MEA‐based electrolyzer for efficient CO production. Briefly, carbon paper (CP)‐supported AuCu (AuCu/CP) particles were prepared by electrodeposition, and their morphology and composition (and hence, the performance for the electrochemical reduction of CO_2_ to CO) were controlled through deposition parameter modulation. Subsequently, one more element was added to prepare AuCuM trimetallic catalysts (M = In, Mo, Fe), and the deposition parameters were controlled to maintain the original morphology for exploring the compositional effect. AuCuIn exhibited the highest catalytic performance, achieving a CO Faradaic efficiency (FE) of 91.4% at an overpotential of 0.49 V. The electrode fabrication procedure for AuCuIn was transferred onto microporous layer (MPL)‐coated CP (MPL/CP), and the composite was directly employed as the cathode of an MEA‐based CO_2_ electrolyzer (Figure S1, Supporting Information). Accordingly, high CO selectivity (CO FE ≈ 100%) and a superior current density (220.1 mA cm^−2^) were achieved at a low operating cell voltage (2.8 V_cell_).

## 2 Results and Discussion

Figures S2–S4, Supporting Information, present the results of preliminary experiments conducted to optimize the deposition potential for AuCu catalyst fabrication. Based on the linear sweep voltammetry curve of CP recorded in the deposition electrolyte (Figure S2, Supporting Information), the deposition potentials were chosen as −0.60, −0.80, and −1.00 V_SCE_ (SCE: saturated calomel electrode). Figure S3, Supporting Information, shows the field‐emission scanning electron microscopy (FESEM) images of pretreated CP and AuCu deposits obtained at the above deposition potentials after 300 s, revealing that these deposits were uniformly formed on the top surface of carbon fibers in all cases. As the deposition potential became increasingly negative, the catalyst surface became rougher, and the Au:Cu ratio increased from 3.25 to 5.05 (Figure S4, Supporting Information) because of the difference in the standard reduction potentials of Au and Cu. The optimal deposition potential was −0.60 V_SCE_, at which the Au:Cu ratio was ≈3.0.^[^ ^29^, ^30^ ^]^

**Figure 1:**
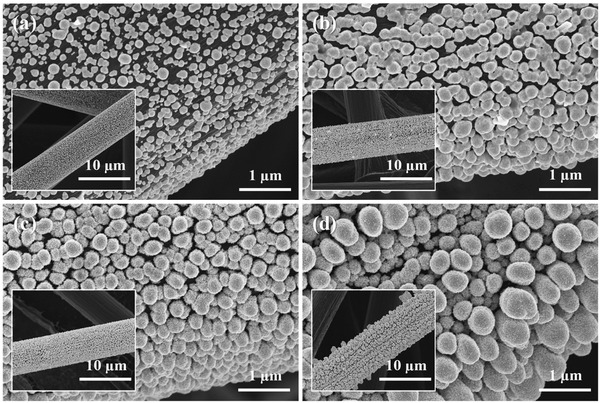
FESEM images of a) AuCu10/CP, b) AuCu50/CP, c) AuCu100/CP, and d) AuCu300/CP. Insets: lower‐magnification FESEM images.

**Figure** **1** shows the FESEM images of AuCu bimetallic catalysts on CP substrate, prepared using a deposition potential of −0.60 V_SCE_ and different deposition times (AuCu#/CP, where “#” represents the deposition time in s). AuCu10/CP comprised sparse spherical particles of different sizes on the carbon fiber surface (Figure 1a), while AuCu50/CP contained more agglomerated spheres (Figure 1b), and AuCu100/CP featured slightly larger dense cauliflower‐like spheres with a rougher surface (Figure 1c). In the case of AuCu300/CP, ellipsoidal particles of different sizes were formed because of growth in the vertical direction (Figure 1d). Despite these morphological differences, the Au:Cu ratios of all samples were similar (2.93–3.25, Figure S5, Supporting Information) and well‐matched with the optimal ratios reported for AuCu catalysts.^[^ ^29^, ^30^, ^31^, ^32^ ^]^

**Figure 2:**
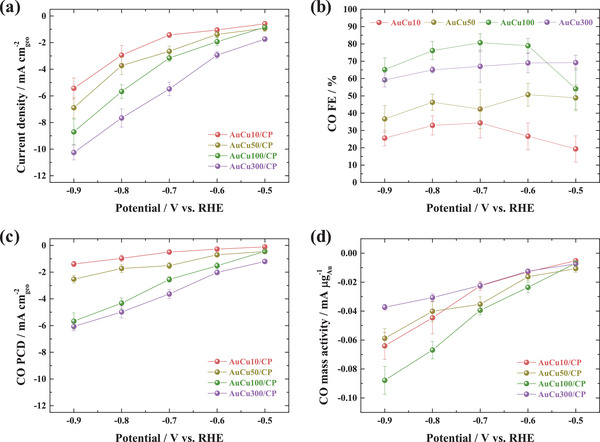
a) Polarization curves, b) CO FE, c) CO PCD, and d) CO mass activity of AuCu#/CP as functions of applied potential.

The catalytic performance of AuCu#/CP was evaluated in the cathode part of an H‐type cell containing CO_2_‐saturated 0.5 m KHCO_3_ as the catholyte. Figure S6, Supporting Information, shows the representative chronoamperometries of AuCu#/CP among three times measurements. **Figure** **2**a shows the average stabilized current densities extracted from the range of 1770–1800 s. In the employed potential range, the magnitude of the current density increased with increasing deposition time because of the concomitant surface roughening and resulting increase in the number of active sites (Figure 1). The gaseous products generated within 1770–1800 s were analyzed by gas chromatography; the CO FE was calculated as a function of the potential (Figure 2b), and typically found to follow the order AuCu100/CP > AuCu300/CP > AuCu50/CP > AuCu10/CP. AuCu100/CP exhibited the highest CO FE of 80.7% (at −0.70 V_RHE_, RHE: reversible hydrogen electrode), showing the highest CO selectivity. In addition, the sum of the CO and H_2_ FEs was ≈100% for all samples in the employed potential range, indicating the absence of other gaseous or liquid products (Figure S7, Supporting Information). Hence, the above catalysts were suitable for the efficient production of syngas with controlled H_2_:CO ratios of 0.18–3.68.^[^ ^51^, ^52^ ^]^ Measurements of the partial current density (PCD) of CO revealed that the highest CO production rate was obtained with AuCu300/CP (Figure 2c) despite its lower CO selectivity (than that of AuCu100/CP), owing to its larger number of active sites. However, according to the CO mass activity (Figure 2d) obtained as the CO PCD per Au mass loading (Table S2, Supporting Information), AuCu100/CP exhibited the highest cost‐effectiveness for CO production. Based on the above results, AuCu100/CP was concluded to be the optimal bimetallic catalyst.

**Figure 3:**
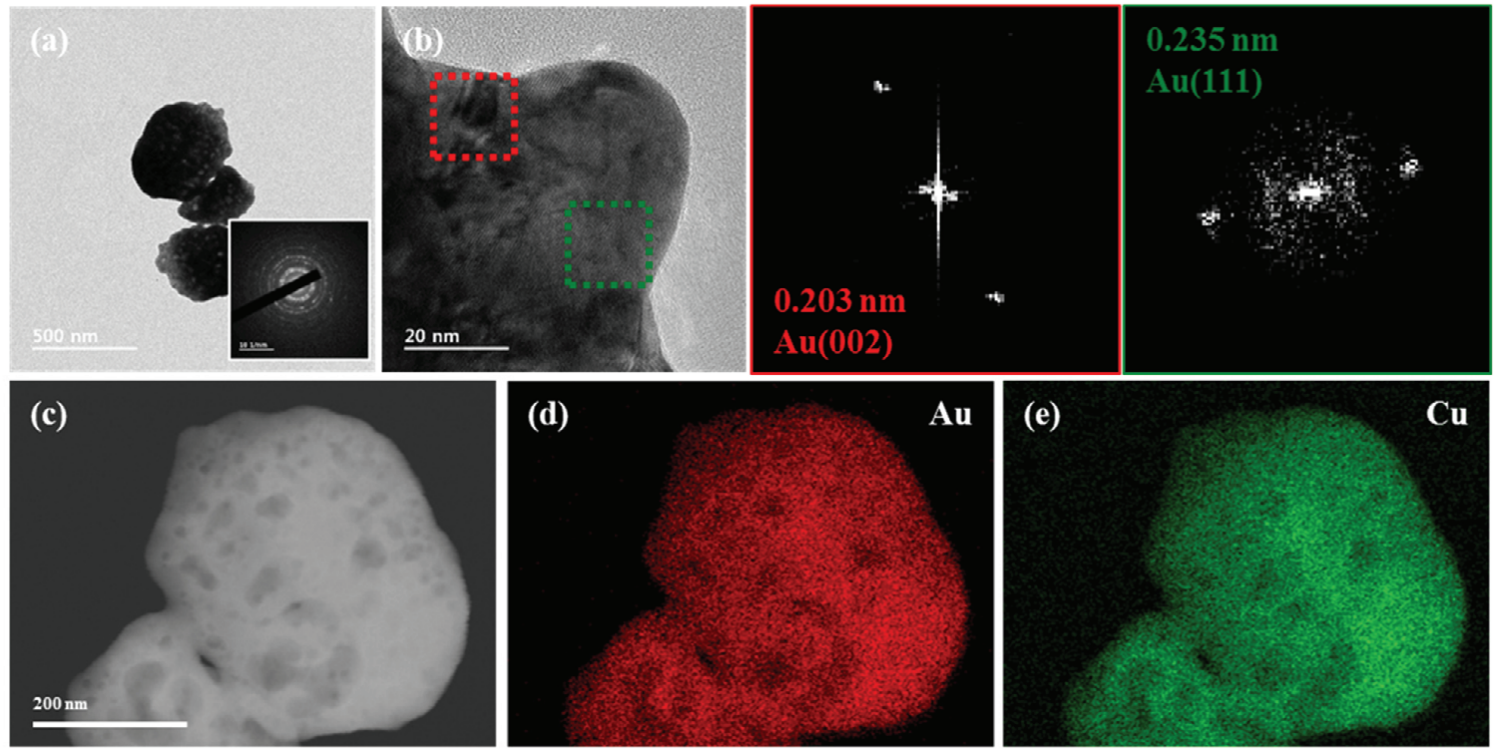
a) TEM image of AuCu100/CP. Inset: SAED pattern. b) HRTEM image of AuCu100/CP with FFT patterns for marked areas. c) Dark‐field TEM image of AuCu100/CP and the corresponding d) Au and e) Cu mappings.

**Figure** **3**a shows a transmission electron microscopy (TEM) image of a AuCu deposit detached from AuCu100/CP. Selected‐area electron diffraction (SAED) analysis confirmed the polycrystalline structure of this deposit, although the diffraction pattern was faint (inset of Figure 3a). The crystal structure of the deposit at the edge sites was analyzed using high‐resolution TEM (HRTEM) and fast Fourier transform (FFT) patterns (Figure 3b and Figure S8, Supporting Information). The red and green boxes indicate the two *d*‐spacings of 0.203 nm (Au (002))^[^ ^53^, ^54^ ^]^ and 0.235 nm (Au (111)),^[^ ^53^, ^54^ ^]^ which were analyzed using GATAN Digital Micrograph software (Figure S9, Supporting Information). The *d*‐spacing values were well‐matched with those of pure Au.^[^ ^53^, ^54^ ^]^ The presence of only two diffracted beam points in each FFT pattern confirmed the low crystallinity of Au in the deposit. Meanwhile, the absence of *d*‐spacings for Cu indicated the presence of amorphous Cu, as confirmed by X‐ray diffraction (XRD) patterns, showing that the weak and broad Au peak appeared without Cu peak (Figure S10, Supporting Information). In Figure 3c, the deposit surface features several voids, which could effectively increase the electrochemical surface area (ECSA). Energy‐dispersive X‐ray spectroscopy (EDS) analysis further showed the uniform distribution of Au and Cu in the deposit (Figure 3d,e). The combined results indicated that AuCu100/CP was a mixture of low‐crystalline Au and amorphous Cu.

**Figure 4:**
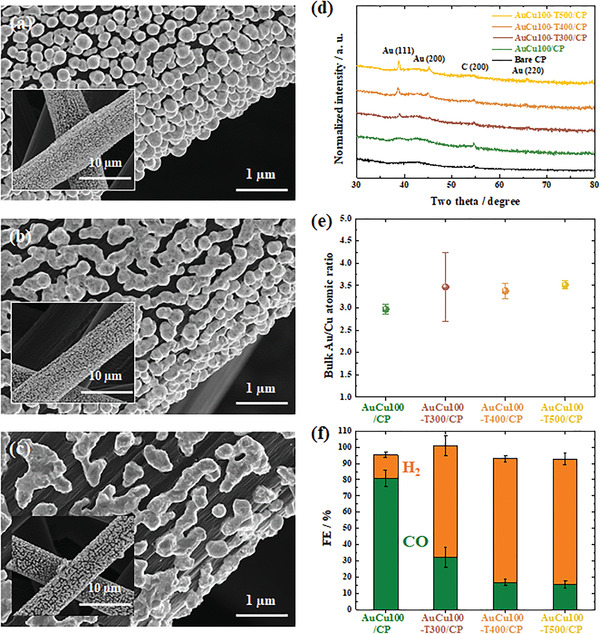
FESEM images of a) AuCu100‐T300/CP, b) AuCu100‐T400/CP, and c) AuCu100‐T500/CP. Insets: lower‐magnification FESEM images. d) XRD patterns, e) Au:Cu atomic bulk ratios, and f) FEs at −0.70 V_RHE_ of AuCu100/CP and AuCu100‐T#/CP.

The low crystallinity of AuCu100/CP was advantageous for catalytic performance enhancement, particularly for selective CO production. Samples obtained after 1 h of annealing at 300, 400, and 500 °C in Ar were denoted as AuCu100‐T#/CP (#: annealing temperature in °C). With increasing annealing temperature, the agglomeration of deposited particles became more severe (**Figure** **4**a–c), and their crystallinity increased, as indicated by the sharpening of XRD peaks (Figure 4d). Compared to those of pure Au, the Au peaks in the patterns of the annealed samples were slightly shifted to higher angles,^[^ ^55^ ^]^ indicating the formation of AuCu alloys. Although the Au:Cu ratios of AuCu100/CP and AuCu100‐T#/CP were similar (3.4–3.5; Figure 4e), the CO FE at −0.70 V_RHE_ significantly decreased with increasing crystallinity (Figure 4f), and so did the intrinsic activity, which was expressed in terms of the scaled current (CO PCD/*C*_dl_) (Figure S11, Supporting Information). The significant decrease in the CO selectivity was ascribed to slower charge transfer in the annealed catalysts, as confirmed by Nyquist (Figure S12a, Supporting Information) and Mott–Schottky (Figure S12b, Supporting Information) plots. This conclusion agreed with previous reports, which revealed that amorphous Cu enables faster charge transfer at catalyst/electrolyte interfaces than crystalline Cu because of the sustained CO_2_ adsorption in the former case.^[^ ^56^ ^]^ A similar enhancement was also observed for amorphous catalysts promoting other electrochemical reactions (e.g., water electrolysis).^[^ ^57^ ^]^ Furthermore, charge transfer was facilitated by the seamless contact between AuCu and CP, as also reported for other self‐supported electrodes.^[^ ^58^ ^]^ Thus, the mixture of low‐crystalline Au and amorphous Cu in AuCu100/CP enhanced the CO selectivity. In addition, the CO FE (80.7% at −0.70 V_RHE_) of AuCu100/CP exceeded the values reported for AuCu alloys with well‐ordered crystalline structures, for example, Au_75_Cu_25_ (64.1%)^[^ ^30^ ^]^ and Au_3_Cu (64.9%).^[^ ^29^ ^]^

**Figure 5:**
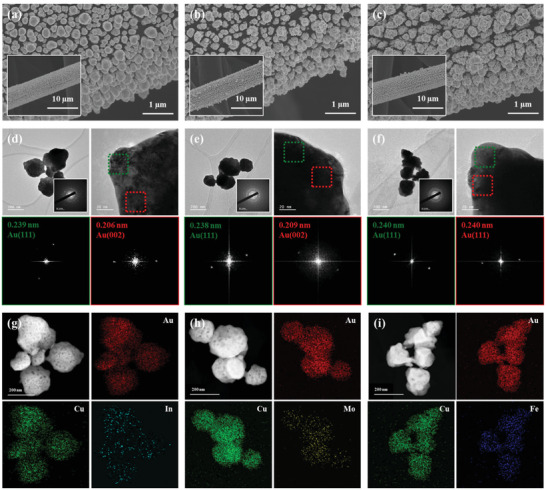
FESEM images of a) AuCuIn/CP, b) AuCuMo/CP, and c) AuCuFe/CP. Insets: low‐magnification FESEM images. HRTEM images of d) AuCuIn/CP, e) AuCuMo/CP, and f) AuCuFe/CP with FFT patterns of the marked areas. Insets: SAED patterns. Dark‐field TEM images and elemental mappings of g) AuCuIn/CP, h) AuCuMo/CP, and i) AuCuFe/CP.

We prepared trimetallic AuCuM/CP electrodes (M = In, Mo, Fe) by electrodeposition using the low‐crystalline bimetallic AuCu/CP electrodes to explore the effect of M on the catalytic performance. The surface composition of the trimetallic electrodes was changed by varying the precursor concentration for third metals in the deposition electrolyte, affecting the CO FEs measured at −0.60 V_RHE_ (Figure S13, Supporting Information). Among them, the deposition parameters were chosen to maintain the Au:Cu ratio (≈3.0) and low crystallinity (Table S1, Supporting Information). Therefore, the properties of AuCuM/CP were mostly similar to those of AuCu100/CP except for the presence of M. FESEM imaging (**Figure** **5**a–c) of AuCuM/CP revealed the presence of agglomerated particles that largely resembled those of AuCu100/CP (Figure 1c) but featured a rougher surface. TEM images and SAED patterns revealed that all AuCuMo/CP had similar particle shapes and polycrystalline structures with faint diffraction patterns (Figure 5d–f). At edge sites, the FFT patterns showed a few diffracted beam points, demonstrating the low crystallinity of Au in AuCuM/CP (see also Figure S14, Supporting Information). In addition, the introduction of M slightly changed the Au *d*‐spacing, for example, the Au (111) *d*‐spacings of AuCuIn/CP, AuCuMo/CP, AuCuFe/CP, and AuCu100/CP equaled 0.239, 0.238, 0.240, and 0.235 nm (Figure 3b), respectively. This change suggested that M was incorporated into the Au lattice during electrodeposition. The corresponding diffraction patterns showed no traces of Cu or M. Thus, the trimetallic AuCuM/CP were concluded to contain mixtures of low‐crystalline Au and amorphous transition metals, and were expected to inherit the advantages of AuCu100/CP. According to the results of dark‐field imaging (Figure 5g–i), the trimetallic catalyst surface featured several voids, which could result in an increased ECSA. Elemental mapping showed a uniform distribution of all elements. The weak signals of M indicated its low content. The atomic surface components of AuCuIn/CP were In (1.7%), Mo (2.8%), and Fe (6.8%), as determined by X‐ray photoelectron spectroscopy (XPS) analysis (Figure S15 and Table S2, Supporting Information). In addition, the position and intensity of the Au 4f and Cu 2p peaks in the core‐level spectra were similar for all AuCuM/CP catalysts.

**Figure 6:**
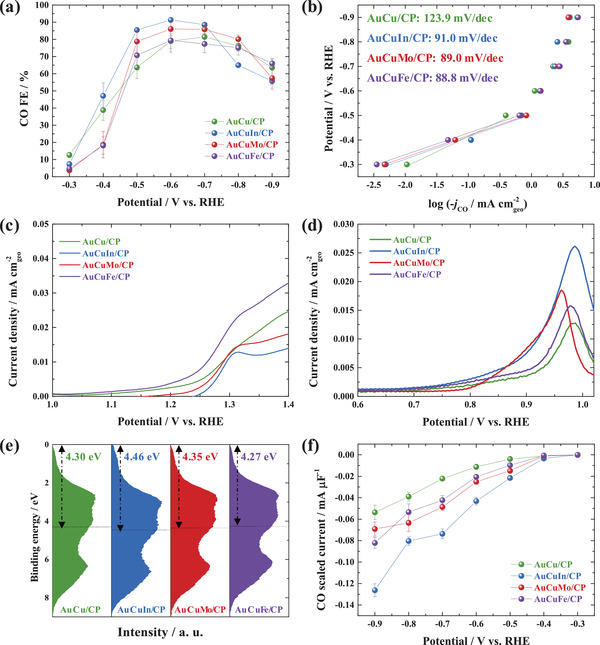
Catalytic performances of AuCu/CP and AuCuM/CP (M = In, Mo, Fe). a) CO FEs, b) Tafel plots derived from CO PCDs, c) OH^−^ adsorption tests, that is, CV curves recorded in N_2_‐purged 0.1 m NaOH at 10 mV s^−1^. d) CO stripping tests, that is, CV curves recorded in CO‐purged 0.5 m KHCO_3_ at 20 mV s^−1^. e) Valence band spectra of AuCu/CP and AuCuM/CP. f) CO scaled currents of AuCu/CP and AuCuM/CP.

The catalytic performance of AuCuM/CP was examined in an H‐type cell containing CO_2_‐saturated 0.5 m KHCO_3_ as the catholyte and compared with that of AuCu/CP. In particular, the average stabilized chronoamperometric current in the range of 1770–1800 s (Figure S16, Supporting Information) was used to obtain the total current density as a function of the applied potential (Figure S17a, Supporting Information), and this dependence was similar for all catalysts in the employed potential range. Meanwhile, the introduction of M enhanced the CO production selectivity. As shown in **Figure** **6**a, the CO FEs of AuCuM/CP mostly exceeded those of AuCu/CP, specifically in the potential range of −0.50 to −0.70 V_RHE_. The sum of the CO and H_2_ FEs was ≈100% for all catalysts in the entire potential range, which confirmed that no other products were formed in significant amounts (Figure S18, Supporting Information). The maximum CO FE (91.4% at −0.60 V_RHE_) was obtained for AuCuIn/CP. Figure 6b shows the Tafel plots derived from CO PCDs (Figure S17b, Supporting Information). The Tafel slope of AuCu/CP (123.9 mV dec^−1^) suggested that the rate‐determining step was the formation of CO_2_^•−^ via initial electron transfer to CO_2_.^[^ ^24^ ^]^ However, AuCuM/CP featured lower Tafel slopes of 88.8–91.0 mV dec^−1^, which indicated that the rate‐determining step was the protonation of CO_2_^•−^ by H^+^ provided by HCO_3_^−^ in the electrolyte.^[^ ^24^ ^]^ Thus, the initial electron transfer in AuCuM/CP was faster than that in AuCu/CP, in line with the order of the corresponding charge transfer resistances extracted from Nyquist plots (Figure S19a, Supporting Information) and that of charge carrier concentrations extracted from Mott–Schottky plots (Figure S19b, Supporting Information). Therefore, the AuCuM/CP surface stabilized CO_2_^•−^ better because of faster electron transfer.

Catalyst surface oxophilicity is an important factor influencing the stability of reaction intermediates (e.g., *COOH and *OCHO).^[^ ^30^, ^59^ ^]^ Herein, the surface oxophilicities of AuCu/CP and AuCuM/CP were determined from the results of cyclic voltammetry (CV) scanning in N_2_‐purged 0.1 m NaOH (Figure S20, Supporting Information), with the positive CV scans presented in Figure 6c. Based on the varied onset potential of OH^−^ adsorption, the surface oxophilicity followed the order of AuCuIn/CP < AuCuMo/CP < AuCu/CP < AuCuFe/CP. Thus, the surface of AuCuIn/CP favored the formation of *COOH over that of *OCHO,^[^ ^60^ ^]^ which benefited selective CO production. Moreover, as the final step of the reaction mechanism, CO desorption is important for selective CO production. Accordingly, the CO binding energy on the catalyst surface was estimated by performing CO stripping tests (Figure 6d and Figure S21, Supporting Information). AuCuIn/CP exhibited the most negative onset potential for CO stripping and hence, the most facile CO desorption. As reported previously,^[^ ^61^ ^]^ the binding energies of reaction intermediates can be controlled by modulation of the surface electronic structure. When the d‐band center moves away from the Fermi level, the antibonding state shifts to lower energies and becomes occupied, thereby weakening intermediate adsorption. Figure 6e shows that the introduction of M shifted the catalyst d‐band center and thus, possibly provided appropriate intermediate binding energies to increase the CO FE.^[^ ^29^, ^30^ ^]^ Moreover, the CO mass activity (CO PCD/Au mass loading) showed that AuCuM/CP was more cost‐effective than AuCu/CP (Figure S22 and Table S2, Supporting Information). For intrinsic activity evaluation, the ECSAs of AuCu/CP and AuCuM/CP were estimated from their double‐layer capacitance (*C*_dl_) values extracted from repeated CV scans performed at various rates in 0.5 m KHCO_3_ (Figure S23, Supporting Information). Figure 6f shows the intrinsic activities expressed as the CO scaled current, revealing that all AuCuM/CP catalysts were more intrinsically active than AuCu/CP in the employed potential range. The highest intrinsic activity was obtained for AuCuIn/CP, which was ascribed to its CO_2_^•−^ stabilization ability, lower surface oxophilicity, and decreased CO binding energy, as described above. Moreover, the addition of In likely suppressed the competitive hydrogen evolution reaction (HER) because of the very weak H binding energy of this element,^[^ ^62^, ^63^ ^]^ as previously reported for bimetallic Ag–In catalysts.^[^ ^64^, ^65^ ^]^

The low crystallinity of AuCuIn/CP also facilitated selective CO production. Samples obtained after 1 h of annealing at 300, 400, and 500 °C in Ar were denoted as AuCuIn‐T#/CP (#: annealing temperature in °C). With increasing annealing temperature, the agglomeration of deposited particles became more severe (Figure S24a–c, Supporting Information), while their crystallinity gradually increased with the sharpening of XRD peaks (Figure S24d, Supporting Information). The CO FE at −0.60 V_RHE_ significantly decreased with increasing crystallinity (Figure S24e, Supporting Information), which decreased the intrinsic activity remarkably (Figure S25, Supporting Information). Thus, the lower CO selectivity of crystalline catalysts originated from their slower charge transfer, as confirmed by analyses of the Nyquist (Figure S26a, Supporting Information) and Mott–Schottky (Figure S26b, Supporting Information) plots.

**Figure 7:**
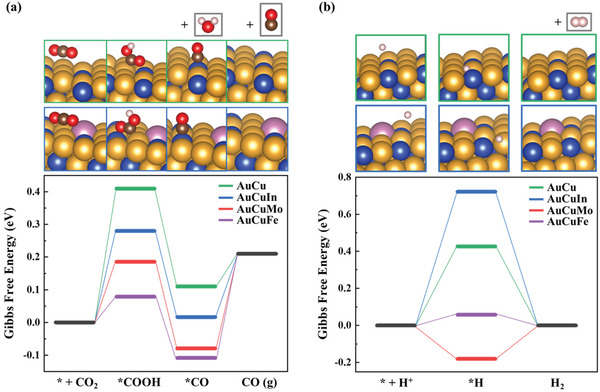
Free energy diagram of a) CO_2_ reduction reaction and b) HER on AuCu and AuCuIn surfaces with geometrical insets. The yellow, blue, purple, brown, red, and white spheres represent Au, Cu, In, C, O, and H atoms, respectively.

In order to obtain an in‐depth mechanistic investigation in atomic scale, the CO_2_ reduction performance of AuCuM was probed using density functional theory (DFT) calculations based on the computational hydrogen electrode (CHE) model.^[^ ^66^ ^]^ **Figure** **7** shows the free energy diagram of the CO_2_ reduction reaction and the HER on AuCu and AuCuM surfaces (insets in Figure 7 show the optimized geometrical structures of AuCu and AuCuIn). The binding energy of COOH (*E*_adh_) was calculated as *E*_adh_ = *E*[*COOH] − (*E*[*] + *E*[COOH]), where *E*[*COOH] and *E*[*] are the electronic energies of the surface with and without adsorbed COOH, respectively; *E*[COOH] is the electronic energy of COOH as a free molecule based on the CHE model.^[^ ^66^ ^]^ All electronic energies were determined by considering solvation effects and free energy corrections. Compared with AuCu catalyst, the binding energies of *COOH on trimetallic catalysts were more negative around 12.9, 32.3, and 22.0 kJ mol^−1^ on AuCuIn, AuCuFe, and AuCuMo surface, respectively. Thus, the introduction of third metal component made *COOH formation more thermodynamically favorable. The selective formation of CO also requires HER inhibition. As shown in Figure 7b, the formation of *H on AuCuIn required the highest energy than that on AuCu, AuCuFe, and AuCuMo. This resulted in the effective suppression of the parasitic HER during CO_2_ reduction on AuCuIn surface. In is a well‐known amphoteric element, hence the acidic or basic behavior of indium is determined by surrounding conditions. Indium has the weakest electronegativity (1.78) compared with Au (2.54) and Cu (1.90), which makes the indium to act as a strong Lewis acid and subsequently it hardly reacts with protons. The intrinsic properties of In strongly promote the selective formation of CO. AuCuMo and AuCuFe require less energy to form *COOH than AuCuIn, however, they suffer from competitive reaction to form hydrogen gas (Figure S27, Supporting Information). The selective reactivity of trimetallic catalysts shows a good agreement with Figure S17, Supporting Information.

**Figure 8:**
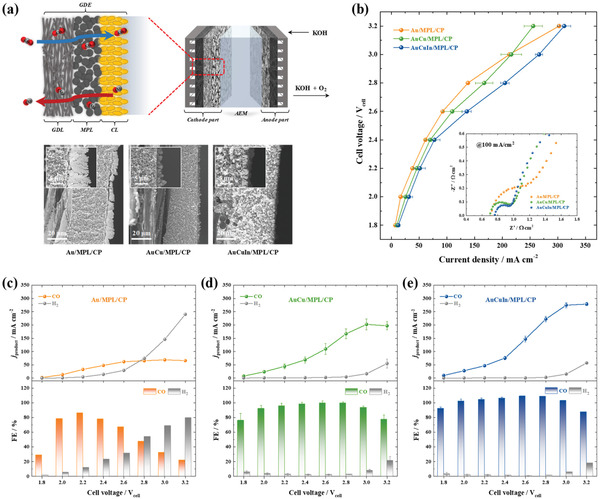
a) Schematic diagram of MEA‐based gaseous CO_2_ electrolyzer and GDE configuration with cross‐sectional FESEM images of Au/MPL/CP, AuCu/MPL/CP, and AuCuIn/MPL/CP cathodes. b) Polarization curves recorded for Au/MPL/CP, AuCu/MPL/CP, and AuCuIn/MPL/CP cathodes. Inset: corresponding Nyquist plots. CO and H_2_ FEs and PCDs as functions of applied cell voltage for c) Au/MPL/CP, d) AuCu/MPL/CP, and e) AuCuIn/MPL/CP cathodes.

The results of catalytic performance evaluation obtained for the conventional CO_2_ electrolyzer and DFT calculations indicated that AuCuIn/CP was the optimal catalyst. As shown in **Figure** **8**a, the cathode of the MEA‐based gaseous CO_2_ electrolyzer was fabricated by the electrodeposition of AuCuIn on MPL/CP to suppress flooding of the gas diffusion layer (GDL) with water as well as to separate the liquid and gas phases during electrochemical CO_2_ reduction.^[^ ^67^ ^]^ Au and AuCu electrodeposited on MPL/CP were used as references. Figure S28, Supporting Information, shows that MPL/CP‐supported AuCu and AuCuIn comprised agglomerated spheres similar to those observed for CP‐supported catalysts (Figures 1c and 5a), whereas MPL/CP‐supported Au exhibited a dendritic morphology. Cross‐sectional FESEM imaging (Figure 8b) showed that porous Au, AuCu, and AuCuIn were vertically grown on the surface of MPL/CP to thicknesses of 2.0–8.1 µm. This would likely generate abundant active sites and reactant pathways, thus maximizing the efficiency of gaseous CO_2_ utilization. EDS analyses of the electrodes showed the uniform distribution of deposited elements on the MPL (Figures S28 and S29, Supporting Information). Furthermore, the detection of F in the MPL indicated the presence of hydrophobic species. XPS analysis revealed that the surface of AuCuIn/MPL/CP contained Au (64.8%), Cu (34.7%), and In (0.5%) (Figure S30, Supporting Information). The MEA comprised an anion‐exchange membrane (AEM); a commercial IrO_2_/CP anode; and MPL/CP‐supported Au, AuCu, or AuCuIn as the cathode. The zero‐gap configuration of this MEA enabled direct contact between the electrodes and AEM, which, together with the absence of a bulk liquid electrolyte, allowed us to minimize the Ohmic resistance.^[^ ^47^ ^]^ Figure 8b shows the polarization curves of MEA‐based gaseous CO_2_ electrolyzers with Au/MPL/CP, AuCu/MPL/CP, and AuCuIn/MPL/CP cathodes. Unlike the conventional CO_2_ electrolyzer, the MEA‐based electrolyzer achieved a current density of hundreds of mA cm^−2^ owing to the much faster diffusion of gaseous CO_2_ compared with that of dissolved CO_2_.^[^ ^39^, ^68^ ^]^ Moreover, the accelerated formation of a TPB at the interface facilitated the realization of high current density, as this TPB provided active sites for gaseous CO_2_.^[^ ^39^, ^50^ ^]^ Figure 8c–e shows FEs and PCDs as functions of the cell voltage. For the Au/MPL/CP cathode, the CO FE increased to ≈85% when the cell voltage increased to 2.2 V_cell_, decreasing at higher cell voltages (Figure 8c). The maximum CO PCD equaled 61.8 mA cm^−2^ at a cell voltage of 2.6 V_cell_. In contrast, the AuCu/MPL/CP cathode exhibited a CO FE of ≈100% in the range of 2.0–2.8 V_cell_, which indicated that highly selective CO production was feasible in a wide cell voltage range (Figure 8d). At cell voltages above 3.0 V_cell_, the CO FE decreased because of the activation of the competitive HER. The maximum CO PCD of 202.3 mA cm^−2^ was achieved at a cell voltage of 3.0 V_cell_. For the AuCuIn/MPL/CP cathode (Figure 8e), similar trends were observed for the CO FE and PCD in a wide range of 1.8–2.8 V_cell_. Nevertheless, the competitive HER remained suppressed at cell voltages above 3.0 V_cell_, which demonstrated the positive effect of In. Consequently, the maximum CO PCD (270.7 mA cm^−2^ at 3.2 V_cell_) of the AuCuIn/MPL/CP cathode exceeded the values obtained for the AuCu/MPL/CP and Au/MPL/CP cathodes by 1.4‐ and 4.1‐fold, respectively. Moreover, the CO PCD of 220.1 mA cm^−2^ at 2.8 V_cell_ and CO FE of ≈100% provided a possibility to satisfy the techno‐economic analysis for the cost of electrochemical CO production (0.6 $ kg^−1^).^[^ ^42^ ^]^ However, it still requires further improvement to be operated in considerably huge current range for realizing commercially available CO_2_ electrolyzer. In addition, the sum of the CO and H_2_ FEs equaled 100% in most voltage ranges, indicating the absence of other products. Meanwhile, the MPL in the GDE prevented flooding of the GDL with water.^[^ ^67^ ^]^ Without the MPL, CP‐supported AuCuIn exhibited much lower performance in the MEA‐based CO_2_ electrolyzer (Figure S31, Supporting Information). In this case, the CO FE decreased from 92.8% to 27.7% when the cell voltage increased from 2.0 to 3.2 V_cell_, with the maximum CO PCD equaling only 45.3 mA cm^−2^ at 3.2 V_cell_. This considerably lower performance was ascribed to the severe flooding of the GDL with water and consequently, the accelerated HER.

**Figure 9:**
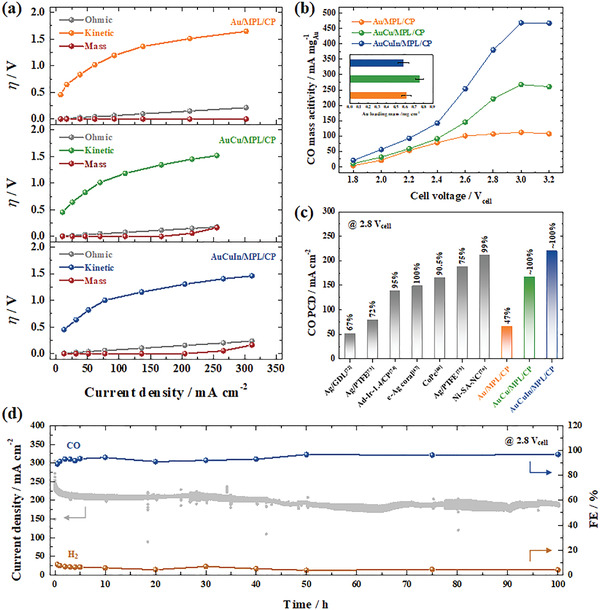
a) Overpotential subdivision analysis derived from the polarization curves of systems with Au/MPL/CP, AuCu/MPL/CP, and AuCuIn/MPL/CP cathodes. b) CO mass activity as a function of cell voltage. Inset: Au mass loadings of Au/MPL/CP, AuCu/MPL/CP, and AuCuIn/MPL/CP cathodes. c) Comparison of CO FEs and PCDs at 2.8 V_cell_ reported in this work with those reported elsewhere for gaseous CO_2_ electrolyzers. d) Long‐term stability test of the AuCuIn/MPL/CP cathode at 2.8 V_cell_ for 100 h.

Based on the polarization curves (Figure 8b), the overpotential was subdivided into three components, namely *η*_ohm_, *η*_kin_, and *η*_mass_ (**Figure** **9**a). The Ohmic resistance of the MEA‐based CO_2_ electrolyzer was 0.698–0.756 Ω cm^2^ based on the corresponding Nyquist plots (Figure S32a,c,e, Supporting Information). The calculated *η*_ohm_ accounted for a small fraction of the total overpotential owing to the direct contact between the electrodes and AEM in the zero‐gap MEA configuration. However, the *η*_kin_ derived from the Tafel plots (Figure S32b,d,f, Supporting Information) accounted for most of the overpotential and increased with increasing current density. The *η*_kin_ and Tafel slope of AuCuIn/MPL/CP were smaller than those of other systems, reflecting the faster reaction kinetics of the former. *η*_mass_, which is related to reactant mass transfer and product emission, was negligibly small at current densities below 200 mA cm^−2^. For the Au/MPL/CP cathode, *η*_mass_ remained negligible at higher current densities because of the considerably slow reaction kinetics of this electrode for CO production. Accordingly, the Tafel plot derived from the H_2_ PCD versus cell voltage curve featured a slope that was mostly constant at all current densities, revealing that the *η*_kin_ of the competitive HER accounted for a large fraction of the total *η*_kin_ (Figure S33a, Supporting Information). Thus, the abundant H_2_O content in the stream of humidified CO_2_ allowed the predominant occurrence of the HER on Au/MPL/CP without *η*_mass_ at a higher current density, resulting in lower selectivity for CO production. The dissociation of H_2_O proceeding in this case afforded protons that were reduced to H_2_ rather than used for CO_2_ reduction.^[^ ^39^ ^]^ In addition, CO_2_ electrolysis at high cell voltages could lower the hydrophobicity of the MPL and thus allow the penetration of H_2_O into the MPL and the generation of H_2_ on carbon species.^[^ ^69^, ^70^, ^71^ ^]^ Meanwhile, for the AuCu/MPL/CP and AuCuIn/MPL/CP cathodes, *η*_mass_ increased at current densities above 200 mA cm^−2^ (Figure 9a). In this range, CO production was limited by the mass transfer of CO_2_, as confirmed by the sharp increase in the Tafel slopes extracted from the CO PCD versus cell voltage curves (Figure S33b,c, Supporting Information). Figure 9b shows the CO mass activity obtained based on the CO PCD and Au mass loading. The AuCuIn/MPL/CP cathode exhibited higher CO mass activity than other cathodes, which demonstrated that the synergetic effects in the trimetallic catalyst resulted in increased cost‐effectiveness. Comparison of the AuCuIn/MPL/CP cathode with previously reported cathodes revealed the superiority of the former in terms of the CO FE and PCD (Figure 9c and Table S3, Supporting Information).^[^ ^47^, ^48^, ^72^, ^73^, ^74^, ^75^, ^76^ ^]^ Furthermore, during the 100 h operation of the AuCuIn/MPL/CP cathode at 2.8 V_cell_, the total current density rapidly decreased in the first 5 h, although the decrease in the next 95 h was very slow. Meanwhile, the CO FE was maintained at ≈100% over the entire 100 h period (Figure 9d). It could be thought that the catalytic properties were mostly maintained in terms of product selectivity; however, the degradation of total current density might be attributed to the mechanical damage on AuCuIn catalysts during the stability test. Further improvement on stability of CO_2_ electrolyzer system still remained a challenge.

## 3 Conclusion

A GDE based on a low‐crystalline trimetallic AuCuIn catalyst exhibited high performance in an MEA‐based gaseous CO_2_ electrolyzer. In the conventional electrolyzer using dissolved CO_2_, this catalyst showed a high CO FE of 91.4% at −0.60 V_RHE_. This was ascribed to its modified electronic structure, fast charge transfer, and suppressed HER originating from the optimal Au:Cu ratio (≈3.0), low crystallinity, and the presence of a third element (In), respectively. Faster electron transfer better stabilized the CO_2_^•−^ intermediate on the AuCuM/CP surface. The low oxophilic surface enabled selective CO production, favoring the formation of *COOH over that of *OCHO, while the downshifted d‐band center facilitated appropriate CO desorption. These advantages were also observed for the MEA‐based gaseous CO_2_ electrolyzer. The AuCuIn/MPL/CP cathode exhibited a CO FE of ≈100% in a wide cell voltage range (2.0–3.0 V_cell_). At a cell voltage of 2.8 V_cell_, the CO PCD reached 220.1 mA cm^−2^ and was superior to values reported previously, providing a possibility to satisfy the techno‐economic requirements for the cost of electrochemical CO production. Thus, our work paves the way to the simple fabrication of high‐performance electrodes offering high CO selectivity, fast CO production, and efficient catalyst utilization. However, further efforts should target the upscaling of the developed strategy using larger MEA areas or electrolyzer stacking for commercial validation.

## 4 Experimental Section

### Preparation of AuCu/CP and AuCuM/CP

AuCu/CP electrodes were prepared using a three‐electrode system comprising a lab‐made Teflon cell with CP as the working electrode, a Pt wire as the counter electrode, and a SCE (KCl saturated) as the reference electrode. The deposition electrolyte contained 5 mm KAuCl_4_·*x*H_2_O (99.99%, Alfa Aesar), 5 mm CuSO_4_·5H_2_O (99%, Daejung), 100 mm KCl (99.5%, Junsei), and 100 mm H_2_SO_4_ (95%, Junsei). Prior to electrodeposition, CP was immersed in 50 wt% HNO_3_ (60–62%, Junsei) at 60 °C for 30 min to increase the hydrophilicity, and all deposition electrolytes were purged by 30 min N_2_ bubbling to remove the dissolved O_2_. Electrodeposition was performed chronoamperometrically using potentiostat (Autolab, PGSTAT302N, Metrohm)‐controlled deposition potentials (−0.60, −0.80, and −1.00 V_SCE_) and deposition times (10–300 s). The prepared AuCu catalysts were annealed in a tube furnace at 300−500 °C for 60 min under Ar.

AuCuM/CP electrodes were prepared by the same method using 5 mm KAuCl_4_·*x*H_2_O (99.99%, Alfa Aesar), 2.5 mm CuSO_4_·5H_2_O (99%, Daejung), and 0.5–20 mm M (M = InCl_3_·*x*H_2_O [99.99%, Alfa Aesar], Na_2_MoO_4_·2H_2_O [98.5%, Daejung], and FeSO_4_·7H_2_O [98%, Daejung]) as precursors. The supporting electrolyte comprised 100 mm KCl (99.5%, Junsei) and 100 mm H_2_SO_4_ (95% Junsei). The deposition potential was fixed at −0.60 V_SCE_ for 100 s. The deposition conditions used for AuCu/CP and AuCuM/CP fabrication are presented in Table S1, Supporting Information.

### Characterization

The surface morphology of the catalyst was analyzed by FESEM (Sigma, Carl Zeiss), while the bulk composition was determined by EDS (Thermo, NORAN System 7). The surface composition and electronic structure were analyzed by XPS (K‐alpha+, Thermo Fisher Scientific), while the crystal structure was examined using XRD (Bruker, D8 Advance). TEM (JEM‐2100 F, JEOL Ltd.) with EDS (Oxford Instruments) was used to investigate the morphology, composition, and crystal structure of the catalyst at high magnification. The catalyst loading was determined by inductively coupled plasma mass spectrometry (NexION300, Perkin Elmer). Specifically, the samples were immersed in a mixture of deionized water (5 mL) and *aqua regia* (5 mL) at 120 °C for 4 h, and the solutions were then diluted with 1% HCl (50 mL) to adjust the concentration to 1, 5, and 10 ng mL^−1^. The loading of each element was determined from the calibration curve constructed using the corresponding standard solution.

Electrochemical characterization was conducted using a lab‐made Teflon cell, with a graphite rod and SCE as the counter and reference electrodes, respectively. The ECSA was estimated from the electrochemical *C*_dl_ determined through repeated CV scanning for each scan rate in N_2_‐purged 0.5 m KHCO_3_ in the open‐circuit potential range of ±0.025 V. The average of current density for the charge and discharge at the open‐circuit potential was plotted as a function of scan rate and then the *C*_dl_ was obtained using the slope. The adsorption/desorption of CO on/from the prepared catalysts was performed in CO‐purged 0.05 m H_2_SO_4_. Specifically, CO adsorption was conducted by applying a potential of 0.10 V_RHE_ for 15 min, and the dissolved CO was then removed by purging the electrolyte with Ar for 20 min. Finally, CO was stripped by CV scanning in the potential range of 0.35–1.15 V_RHE_ at 20 mV s^−1^. The affinity of hydroxide ion adsorption on catalysts was examined in 0.1 m NaOH upon CV scanning in the potential range of 1.00–1.60 V_RHE_ at 10 mV s^−1^.

### Catalytic Performance in Conventional CO_2_ Electrolyzer

The catalytic performance was tested in a lab‐made H‐type cell. A Nafion membrane (212, Dupont Co.) was used to separate the cathode and anode parts. The cathode part contained working and SCE reference electrodes, whereas the anode part contained a Pt mesh counter electrode. The catholyte and anolyte were CO_2_‐ and N_2_‐purged 0.5 m KHCO_3_, respectively. Electrochemical CO_2_ reduction was performed chronoamperometrically at constant potentials in the range from −0.30 to −0.90 V_RHE_ at an interval of 0.10 V for each 30 min. During the reduction, CO_2_ was continuously injected into the catholyte at a flow rate of 12 mL min^−1^ maintained using a mass flow controller (MKS Instruments Inc.). The rate of gas production at the outlet of the cathode part was measured using a flow meter (G6691A, Agilent). The concentrations of CO and H_2_ in the produced gas were determined by gas chromatography (Agilent 7890B) using flame ionization and thermal conductivity detectors, respectively. The FE was calculated from CO and H_2_ concentrations and the related electrical charges, following the equation: *FE*_i_ = *n*_i_ *nF*/*Q*, where *n*_i_ was the moles of product; *n* was the number of electrons used for the formation of one mole of products (*n* = 2 here); *F* was Faraday constant (94,685 C mol^−1^); and *Q* was the total charge in Coulomb passed across the electrode.

### Catalytic Performance in MEA‐Based CO_2_ Electrolyzer

MPL/CP‐supported AuCuIn prepared by electrodeposition was used as the cathode material. In the three‐electrode system with the deposition electrolyte, the deposition potential was fixed at −0.60 V_SCE_ for 1800 s. MPL/CP‐supported Au and AuCu were also prepared by electrodeposition as references. Commercial IrO_2_/CP (C0206, Dioxide Materials) was used as the anode material. A commercial AEM (Sustainion X37‐50, Dioxide Materials) was placed between the cathode and anode to fabricate a zero‐gap MEA. The active area equaled 1 cm × 1 cm, while the cathode and anode gasket thicknesses were 200 µm. Humidified CO_2_ gas was injected into the back side of the cathode at 200 mL min^−1^, while the anode part was supplemented with 0.1 m KOH at 20 mL min^−1^. The electrolyzer was operated at 25 °C. The details for preparation and operation of MEA‐based CO_2_ electrolyzer system are visualized in Figure S1, Supporting Information.

The catalytic performance was determined chronoamperometrically in the potential range of 1.8–3.2 V_cell_ at an interval of 0.2 V for each 15 min. The outlet of the cathode part was directly connected to the gas chromatography for measuring the concentrations of CO and H_2_ in the produced gas. The CO and H_2_ concentrations and the related electrical charges were used to calculate the FE.

For overpotential analysis, the Ohmic resistance was evaluated using electrochemical impedance spectroscopy (Autolab FRA32M, Metrohm). The Ohmic overpotential (*η*_ohm_) was obtained as *η*_ohm_ = *iR*_ohm_, and the polarization curves were *iR*‐corrected. The kinetic overpotential (*η*_kin_) was determined from the Tafel plots of *iR*‐corrected polarization curves. The mass‐transfer overpotential (*η*_mass_) was determined as the remaining overpotential, namely as *η*_mass_ = *E* − *E*_0_ − *η*_ohm_ − *η*_kin_, where *E_0_* denoted the theoretical potential at 25 °C.

### Computational Details for Mechanistic Study

Electronic structures were calculated using the Vienna ab initio simulation package based on Perdew–Burke–Ernzerhof exchange‐correlation functional and projector‐augmented wave pseudopotential.^[^ ^77^ ^]^ AuCu (111) and AuCuIn (111) surfaces were modeled using a 2 × 2 × 2 periodic cell with an 18 Å vacuum layer. The AuCuIn structure was optimized by replacing one Au atom on the top layer of Au_3_Cu_1_ with an In atom, and a structural relaxation with an vacuum layer was followed by slab energy calculation. The AuCuIn slab contained Au (74%), Cu (25%), and In (1%), in line with the experimental conditions. The AuCuMo and AuCuFe slab were formed by replacing In as a Mo and Fe. For all geometry optimizations, the upper three layers of slabs and adsorbates were relaxed until the residual force was less than 0.05 eV Å^−1^ using a cut‐off energy of 400 eV and a 3 × 3 × 1 Monkhorst–Pack mesh. The Gibbs free energy was calculated from the electronic energy by considering solvation effects and free energy corrections (i.e., zero‐point energy, enthalpy, and entropy) using the CHE model.^[^ ^66^ ^]^

## Conflict of Interest

The authors declare no conflict of interest.

## Supporting information

Supporting Information

## Data Availability

The data that support the findings of this study are available from the corresponding author upon reasonable request.
