# Supplementary material for: Low‐Crystalline AuCuIn Catalyst for Gaseous CO2 Electrolyzer
Source: Adv Sci (Weinh). 2022 Jan 22;9(8):2104908. doi: 10.1002/advs.202104908 (PMC8922131; doi:10.1002/advs.202104908)
Supplement: Supplementary file 1 — Supporting Information [file ADVS-9-2104908-s001.pdf]

ADVANCED SCIENCE
Open Access

Supporting Information

for *Adv. Sci.*, DOI 10.1002/advs.202104908

Low-Crystalline AuCuIn Catalyst for Gaseous CO_2 Electrolyzer

Gyeong Ho Han, Junhyeong Kim, Seohyeon Jang, Hyunki Kim, Wenwu Guo, Seokjin Hong, Junhyeop Shin, Inho Nam, Ho Won Jang, Soo Young Kim* and Sang Hyun Ahn**

ADVANCED SCIENCE
Open Access

Supporting Information

for *Adv. Sci.,* DOI: 10.1002/advs.202104908

Low-crystalline AuCuIn catalyst for gaseous CO_2 electrolyzer

Gyeong Ho Han, Junhyeong Kim, Seohyeon Jang, Hyunki Kim, Wenwu Guo, Seokjin Hong, Junhyeop Shin, Inho Nam, Ho Won Jang, Soo Young Kim* and Sang Hyun Ahn**

Supporting Information

Low-crystalline AuCuIn catalyst for gaseous CO_2 electrolyzer

Gyeong Ho Han,† Junhyeong Kim,† Seohyeon Jang, Hyunki Kim, Wenwu Guo, Seokjin Hong, Junhyeop Shin, Inho Nam, Ho Won Jang, Soo Young Kim* and Sang Hyun Ahn**

†These authors contributed equally.

*Corresponding authors

Table S1. Deposition conditions used for the fabrication of bi- and trimetallic electrodes.

Electrodes	Deposition electrolyte configuration					Deposition potential / V_{SCE}	Deposition time / s
	$KAuCl_4 \cdot xH_2O$ / mM	$CuSO_4 \cdot 5H_2O$ / mM	$InCl_3 \cdot xH_2O$ / mM	$Na_2MoO_4 \cdot 2H_2O$ / mM	$FeSO_4 \cdot 7H_2O$ / mM		
AuCu10/CP	5	5	-	-	-	-0.60	10
AuCu50/CP	5	5	-	-	-	-0.60	50
AuCu100/CP	5	5	-	-	-	-0.60	100
AuCu300/CP	5	5	-	-	-	-0.60	300
AuCuIn/CP	5	2.5	0.5, 0.75, 1.25, 10, 20	-	-	-0.60	100
AuCuMo/CP	5	2.5	-	0.75, 1.25, 10, 20	-	-0.60	100
AuCuFe/CP	5	2.5	-	-	0.75, 1.25, 10	-0.60	100

Table S2. Metal loadings and area ratios of XPS peaks for bi- and trimetallic electrodes.

Electrodes	Mass loading / μg cm^{-2}		XPS peak area ratio				
	Au	Cu	Au	Cu	In	Mo	Fe
AuCu10/CP	21.7	0.9	0.597	0.403	-	-	-
AuCu50/CP	43.0	5.0	0.719	0.281	-	-	-
AuCu100/CP	64.6	5.6	0.705	0.295	-	-	-
AuCu300/CP	162.6	15.4	0.639	0.361	-	-	-
AuCuIn/CP	54.1	3.6	0.715	0.268	0.017	-	-
AuCuMo/CP	52.2	1.6	0.685	0.287	-	0.028	-
AuCuFe/CP	67.2	3.7	0.771	0.161	-	-	0.068

Table S3. Configuration, operating conditions, and performance of state-of-the-art MEA-based CO_2 electrolyzer.

Cathode	Anode	Membrane	MEA type	Electrode area / cm^2	CO_2 flow rate / mL min^{-1}	Anolyte	CO PCD @ 2.8 V_{cell} / mA cm^{-2}	FE @ 2.8 V_{cell} / %	Reference
Ag/GDL (1.5 mg cm^{-2})	Ni foam	BPM	CCS	4	100	1.0 M NaOH	50.9	67	72
Ag/PTFE (-)	Ni foam	AEM FAA-3-PK-130	CCM	0.27	50	1.0 M KOH	78.5	72	73
Ad-Ir-1.4/CP (1.0 mg cm^{-2})	IrO_2/Ti	AEM FAA-3-PE-30	CCS	-	50	0.5 M KOH	137.7	95	74
Ag coral (0.5 mg cm^{-2})	IrO_2/GDL	AEM X37-50	CCS	10	100	1.0 M KOH	148.0	100	47
CoPc (0.2 mg cm^{-2})	IrO_2/GDL	APE	CCS	3.2	100	Deionized water	165.6	90.5	48
Ag/PTFE (1.0 mg cm^{-2})	Ni foam	AEM X37-50	CCS	4	100	1.0 M KOH	187.5	75	75
Ni-SA-NC (0.3 mg cm^{-2})	Ir/C	AEM X37-50	CCS	1	-	0.5 M $KHCO_3$	210.9	99	76
Au/MPL/CP (0.613 mg cm^{-2})	IrO_2/C/CP	AEM X37-50	CCS	1	200	0.1 M KOH	65.4	47	This work
AuCu/MPL/CP (0.755 mg cm^{-2})	IrO_2/C/CP	AEM X37-50	CCS	1	200	0.1 M KOH	167.0	100	This work
AuCuIn/MPL/CP (0.579 mg cm^{-2})	IrO_2/C/CP	AEM X37-50	CCS	1	200	0.1 M KOH	220.1	100	This work

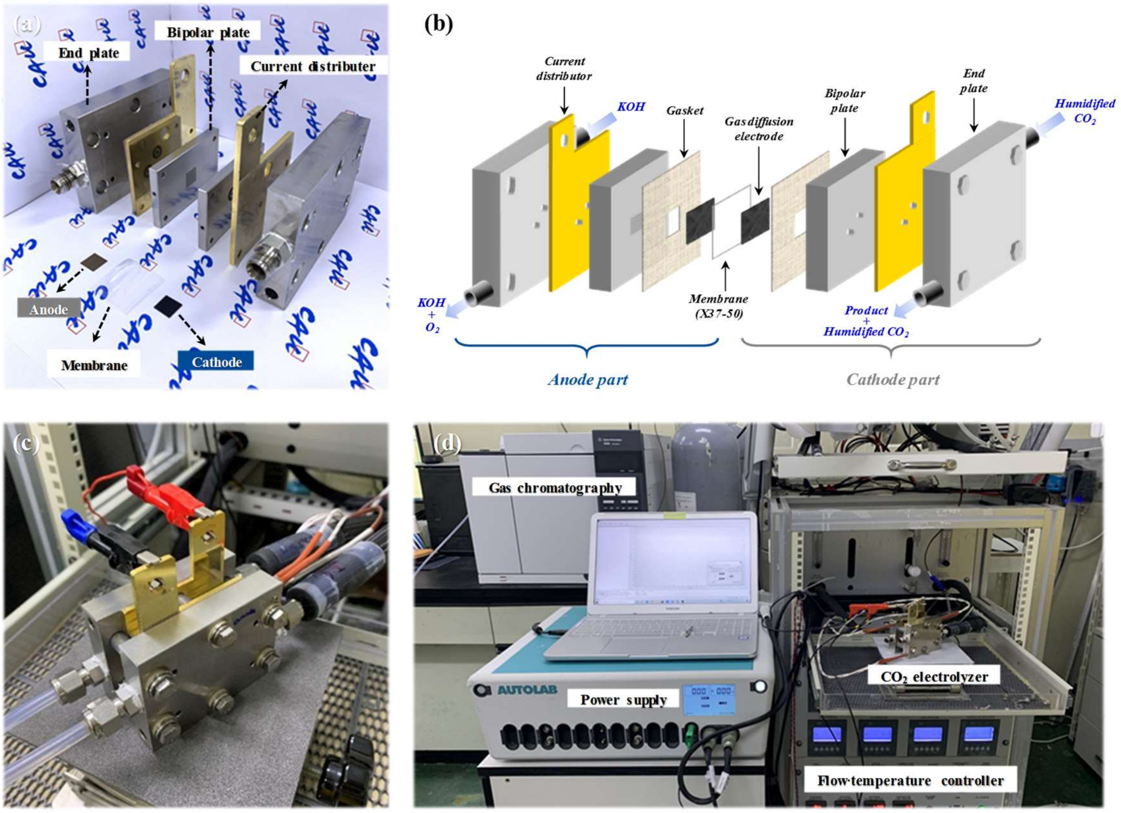

Figure S1. (a) Photo image and (b) schematic illustration of MEA-based CO_2 electrolyzer configuration. (c) Photo images of assembled MEA-based CO_2 electrolyzer and (d) performance analysis system.

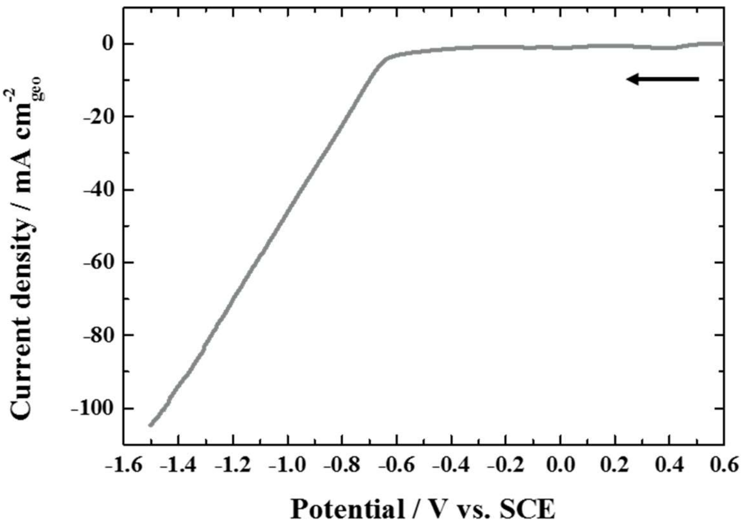

Figure S2. Linear sweep voltammogram of bare CP recorded in N_2-purged deposition electrolyte (5 mM $KAuCl_4$, 5 mM $CuSO_4$, 100 mM KCl, and 100 mM H_2SO_4) at 10 mV s^{-1}.

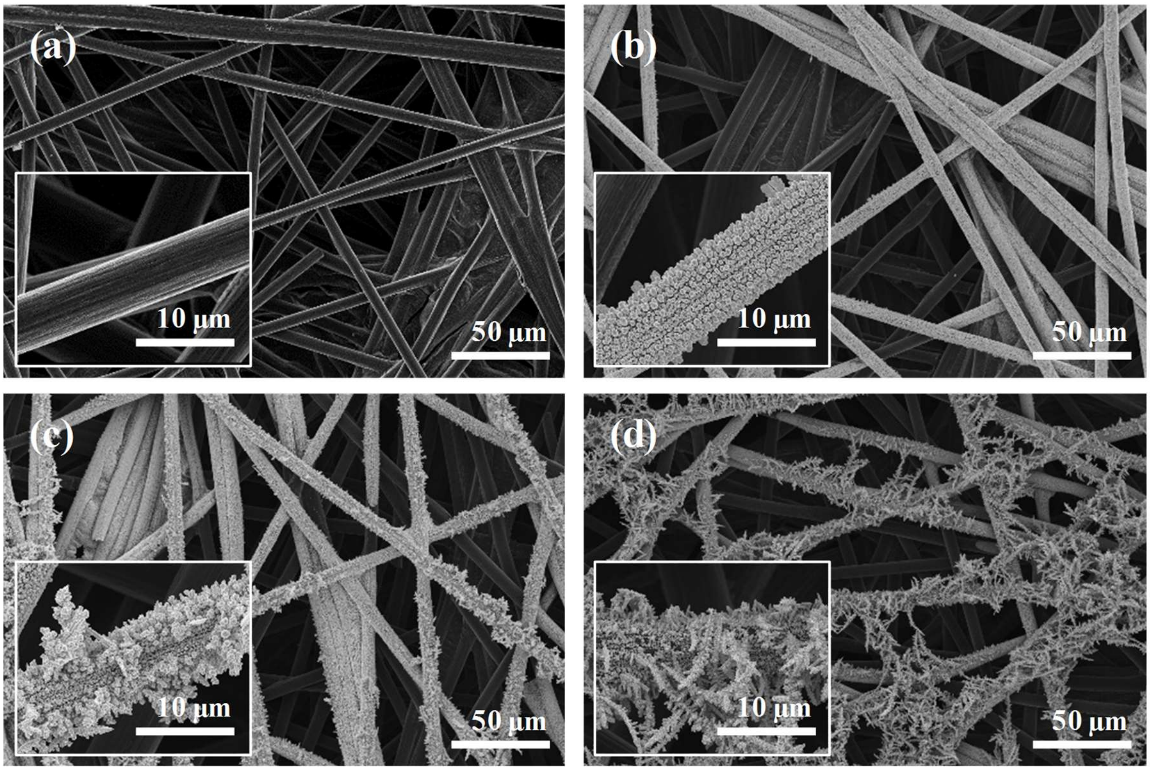

Figure S3. FESEM images of (a) bare CP and CP-supported AuCu prepared at deposition potentials of (b) −0.60, (c) −0.80, and (d) −1.00 V_{SCE} for 300 s. Insets show higher-magnification FESEM images.

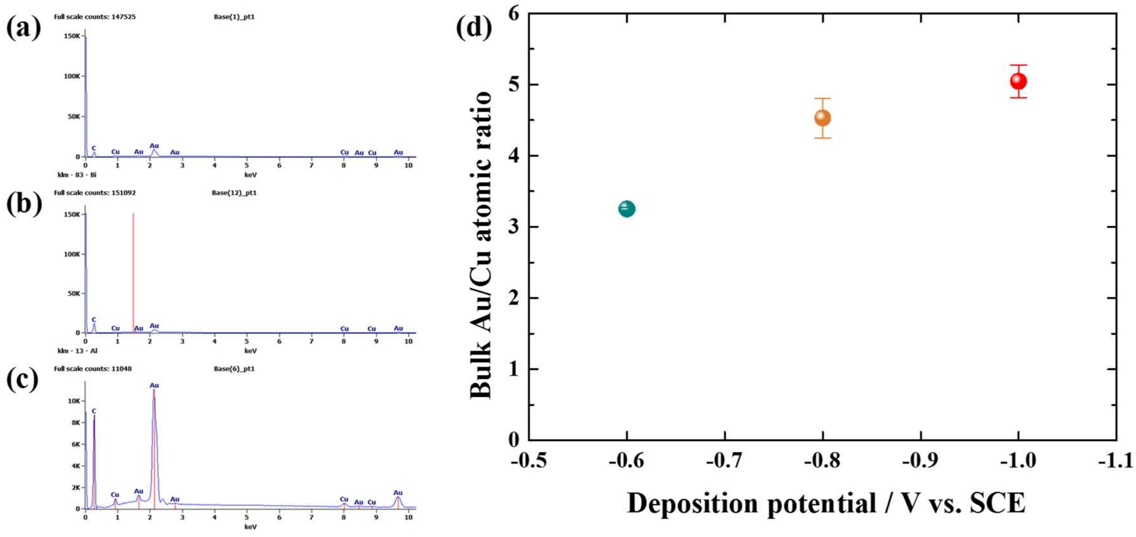

Figure S4. EDS profiles of CP-supported AuCu prepared at deposition potentials of (a) −0.60, (b) −0.80, and (c) −1.00 V_{SCE} for 300 s. (d) Effect of deposition potential on the bulk Au:Cu atomic ratio.

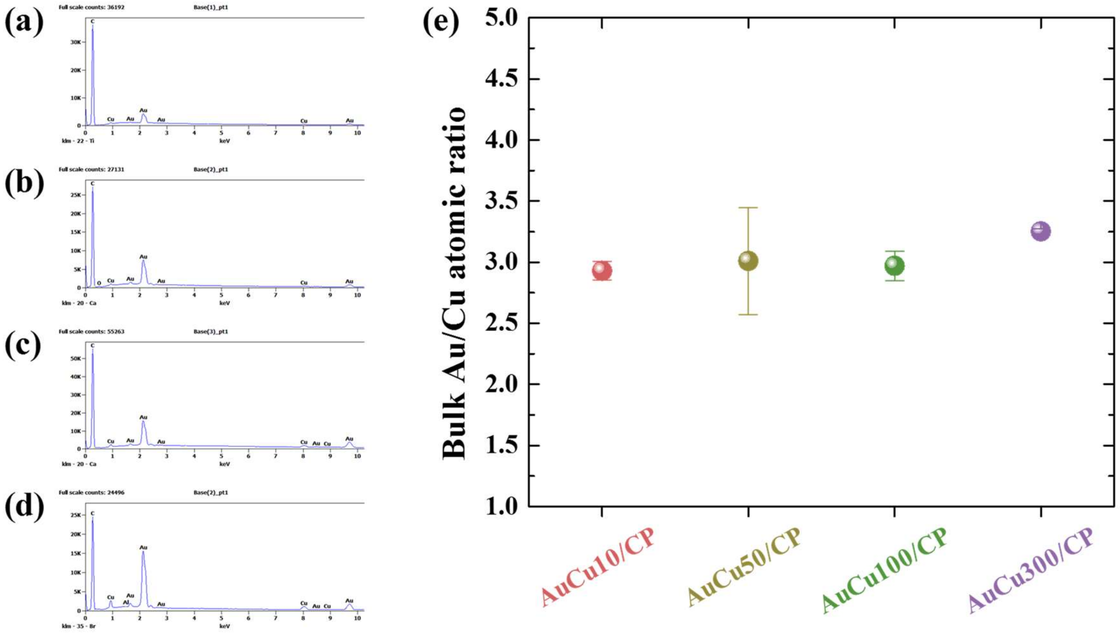

Figure S5. EDS profiles of (a) AuCu10/CP, (b) AuCu50/CP, (c) AuCu100/CP, and (d) AuCu300/CP prepared at a deposition potential of −0.60 V_{SCE} using different deposition times. (e) Effect of deposition time on the bulk Au:Cu atomic ratio.

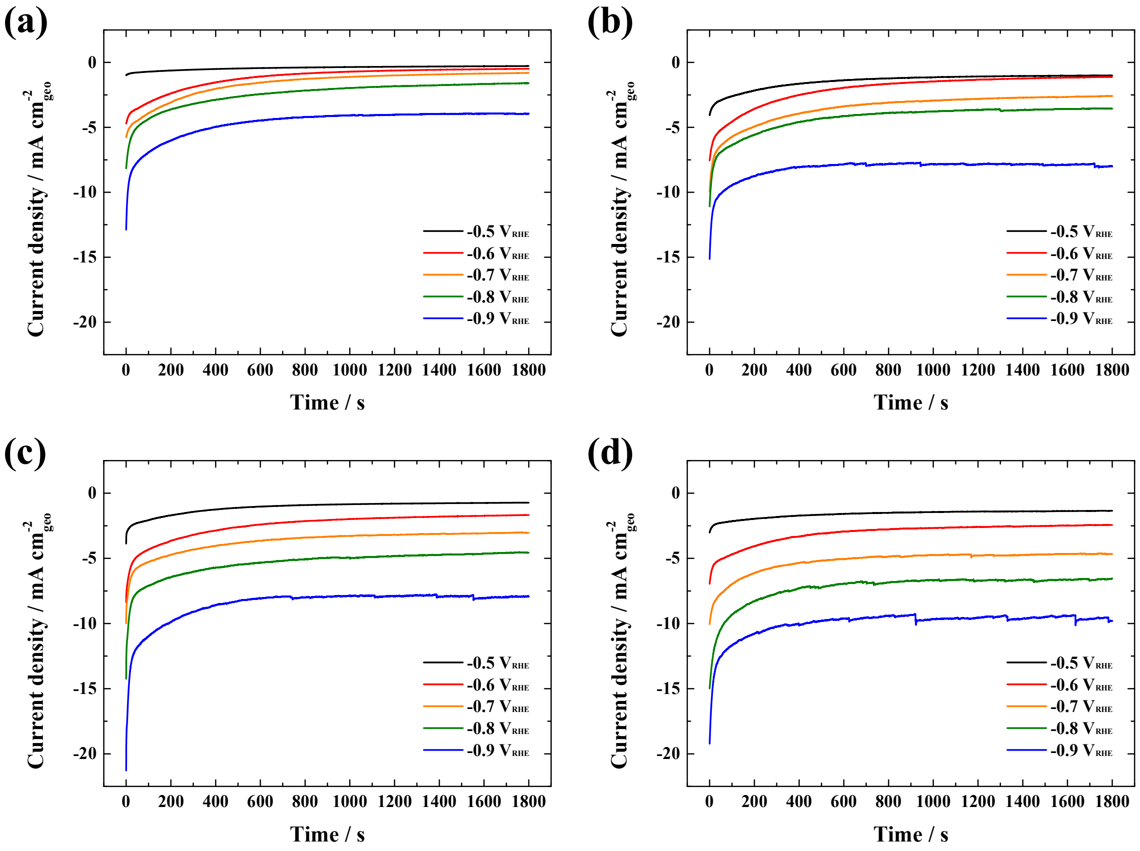

Figure S6. Representative chronoamperometric curves recorded at different applied potentials in CO_2-saturated 0.5 M $KHCO_3$ for (a) AuCu10/CP, (b) AuCu50/CP, (c) AuCu100/CP, and (d) AuCu300/CP.

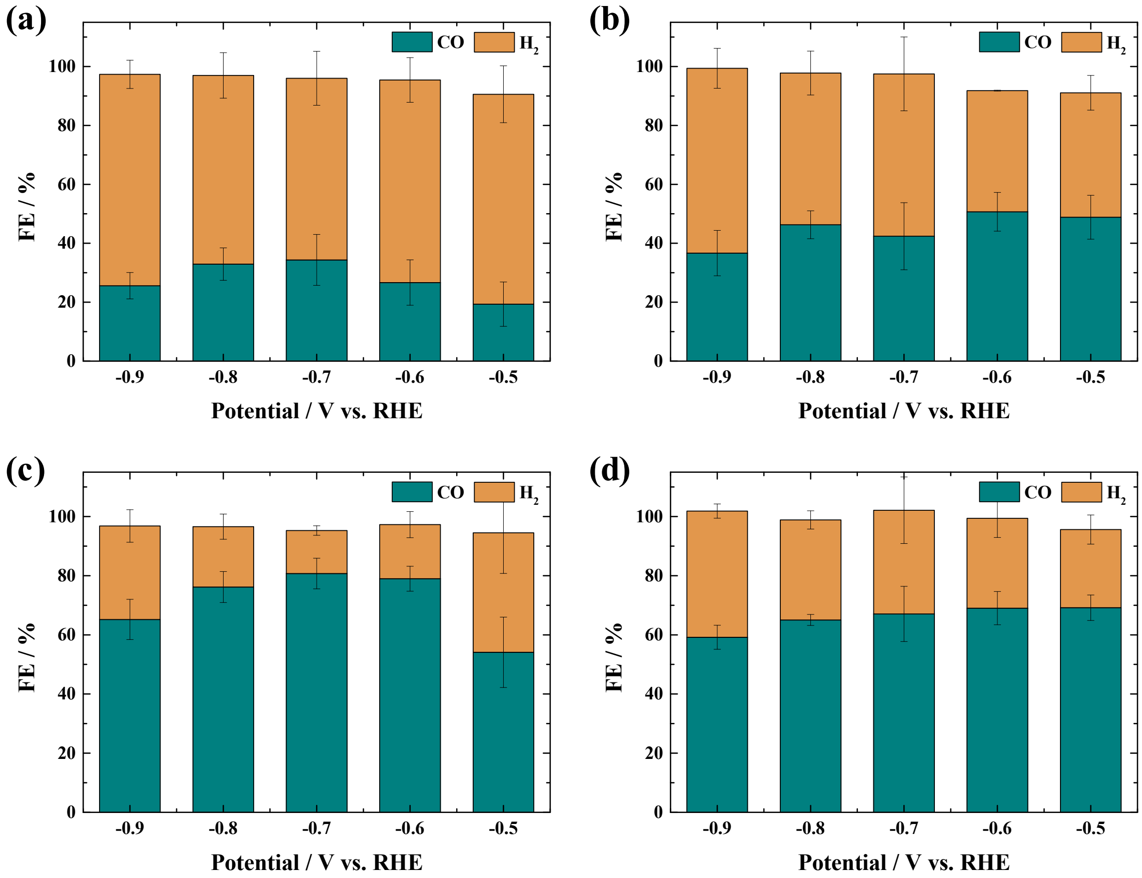

Figure S7. Sum of CO and H_2 FEs as a function of applied potential for (a) AuCu10/CP, (b) AuCu50/CP, (c) AuCu100/CP, and (d) AuCu300/CP.

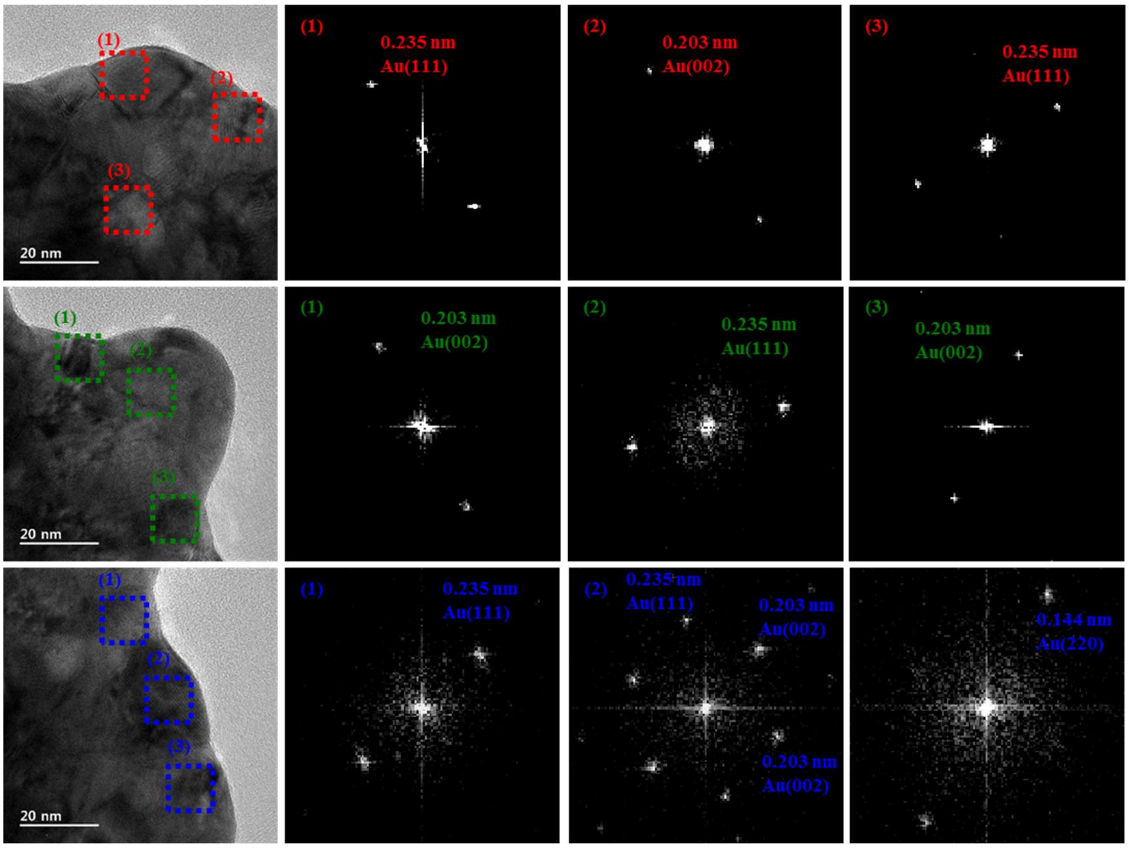

Figure S8. HRTEM images of AuCu100/CP and corresponding FFT patterns of marked areas.

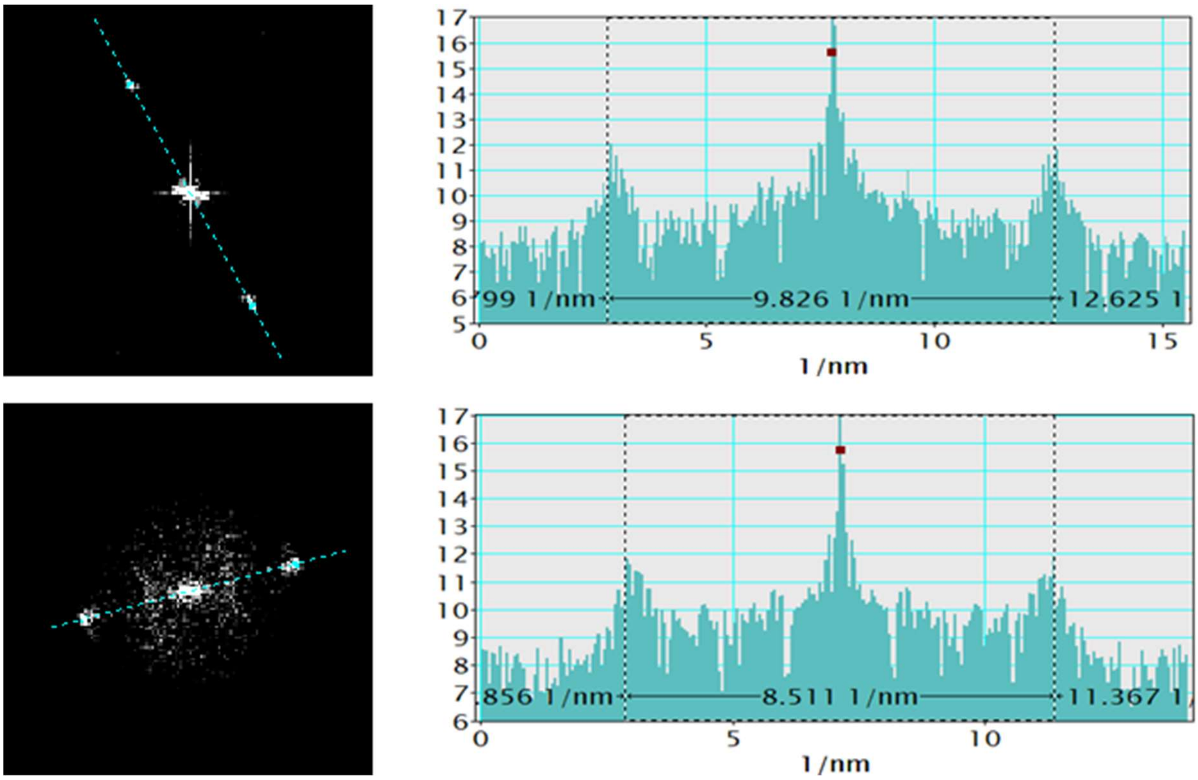

Figure S9. Analysis of the FFT patterns of AuCu100/CP by GATAN Digital Micrograph software.

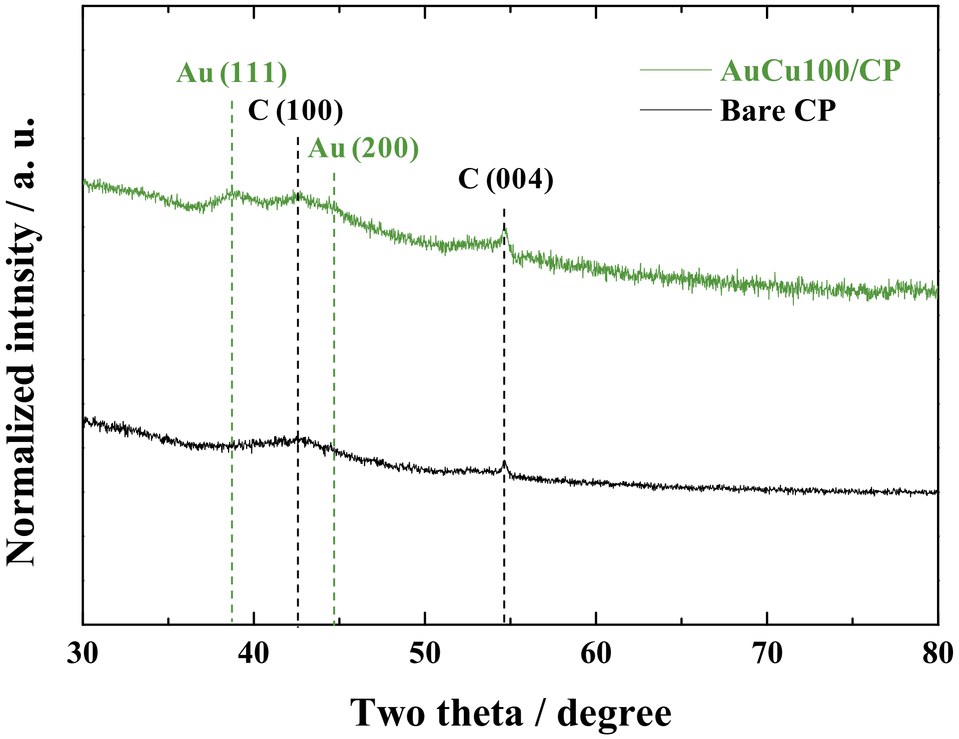

Figure S10. Normalized-intensity XRD patterns of bare CP and AuCu100/CP.

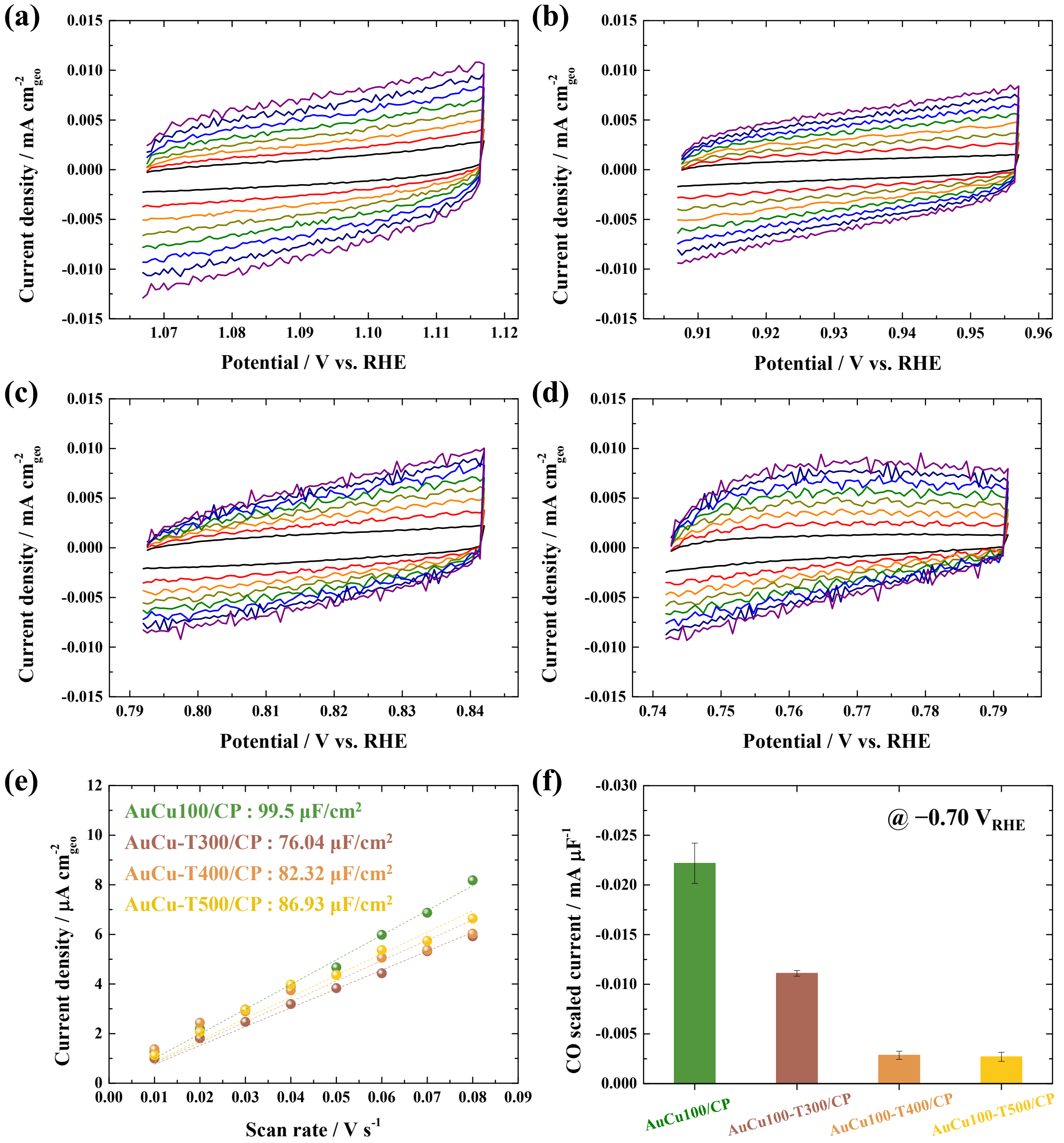

Figure S11. Repeated CV curves recorded at 10–80 mV s^{-1} in N_2-purged 0.5 M $KHCO_3$ for (a) AuCu100/CP, (b) AuCu-T300/CP, (c) AuCu-T400/CP, and (d) AuCu-T500/CP. (e) Results of C_{dl} measurements and (f) CO scaled currents at –0.70 V_{RHE} obtained for AuCu100/CP and AuCu-T#/CP.

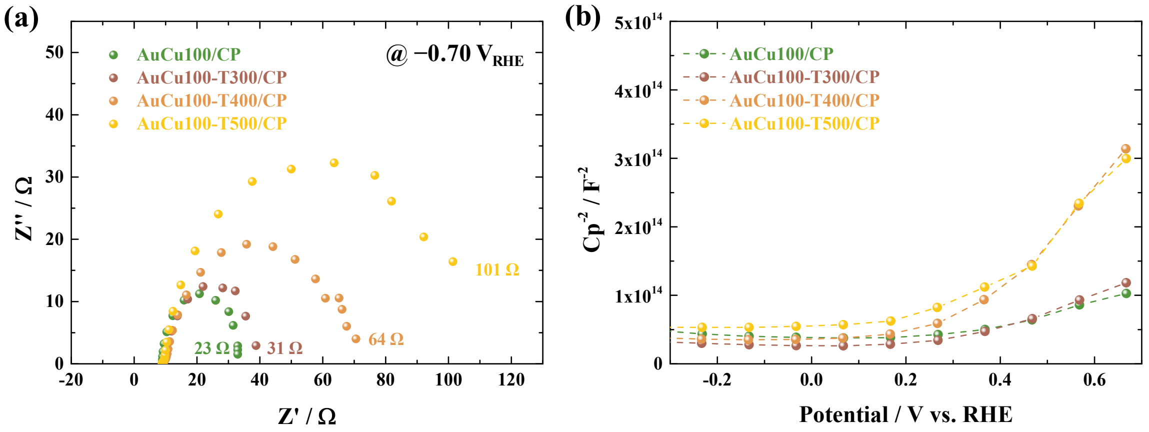

Figure S12. (a) Nyquist plots at −0.70 V_{RHE} and (b) Mott–Schottky plots of AuCu100/CP and AuCu-T#/CP.

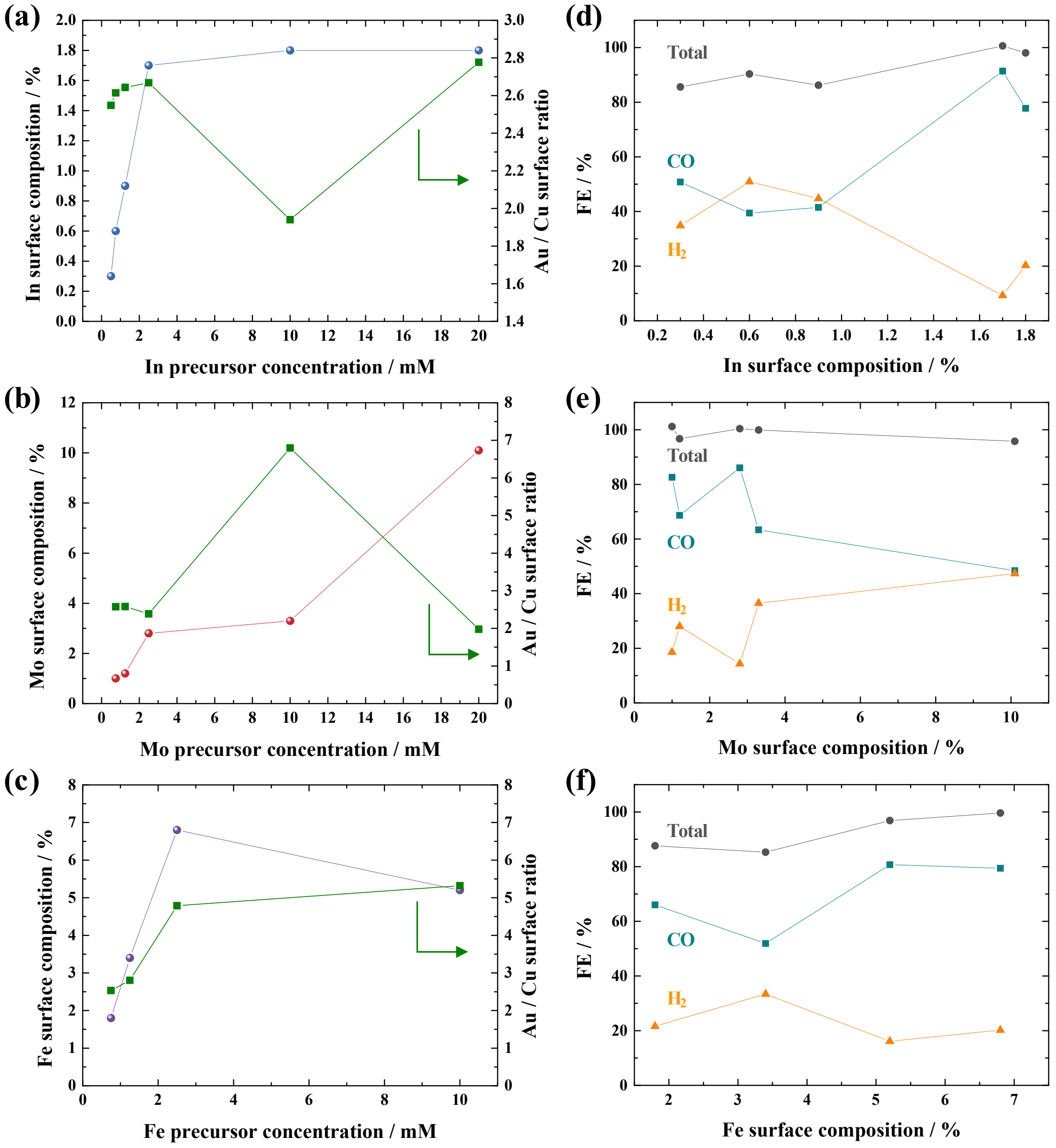

Figure S13. (a-c) Surface composition of trimetallic electrodes depending on precursor concentration of third metals in the deposition electrolytes for (a) AuCuIn/CP, (b) AuCuMo/CP, and (c) AuCuFe/CP. (d-f) FEs measured at −0.6 V_{RHE} depending on surface composition of third metals for (d) AuCuIn/CP, (e) AuCuMo/CP, and (f) AuCuFe/CP.

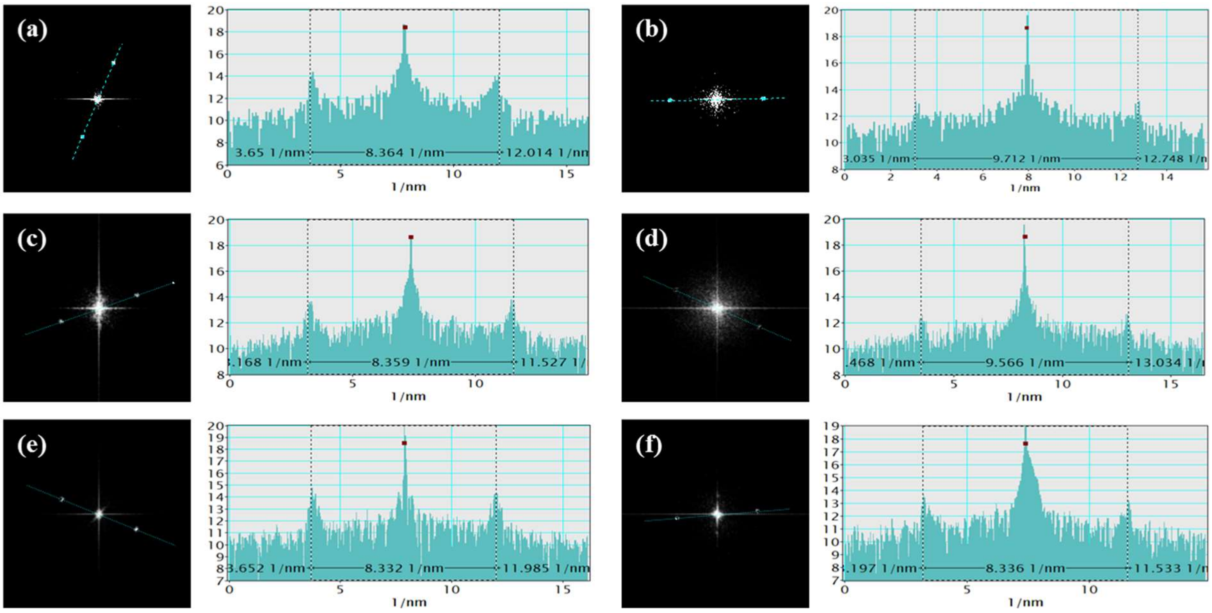

Figure S14. Analysis of the FFT patterns of (a, b) AuCuIn/CP, (c, d) AuCuMo/CP, and (e, f) AuCuFe/CP using GATAN Digital Micrograph software.

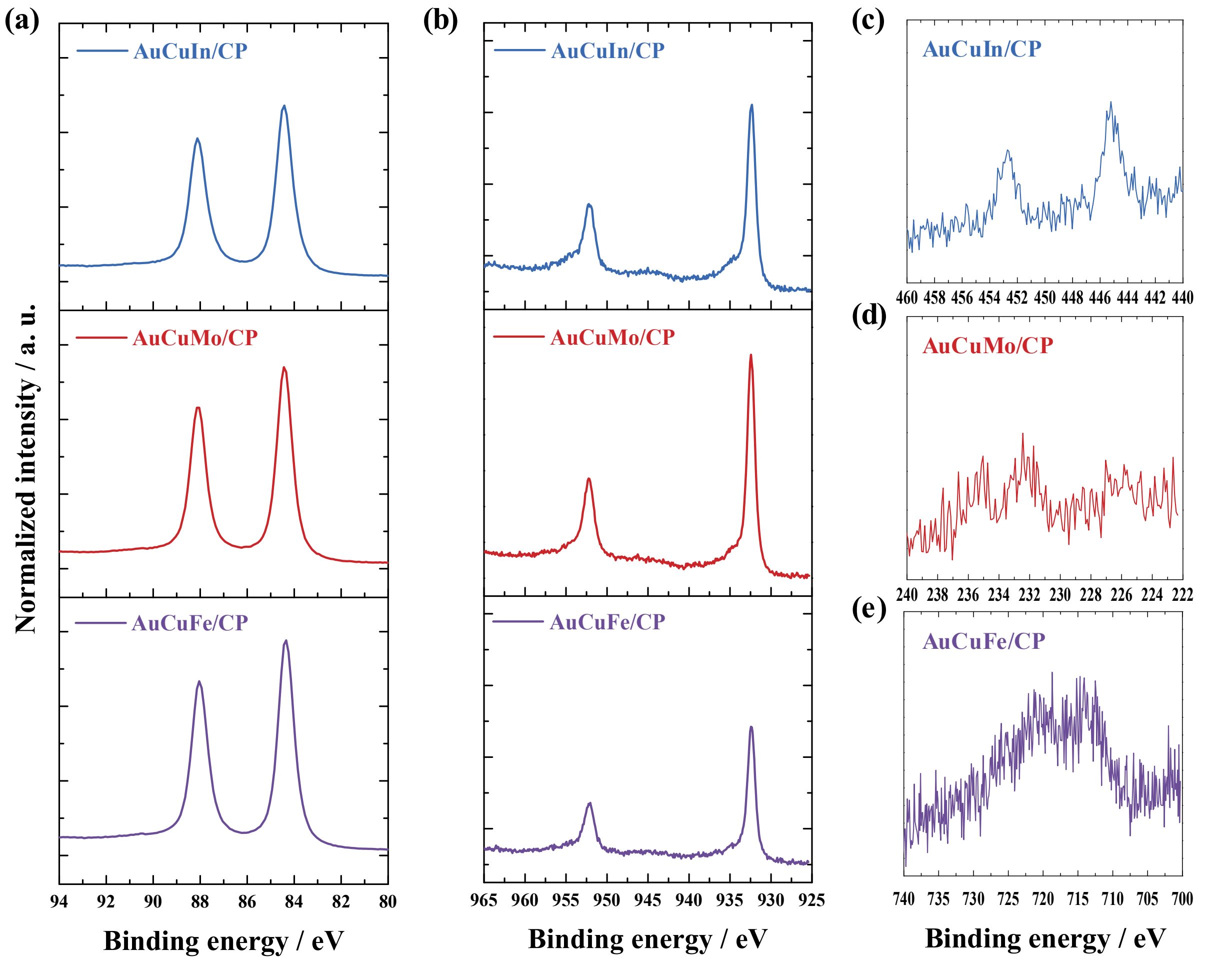

Figure S15. (a) Au 4f and (b) Cu 2p spectra of AuCuIn/CP, AuCuMo/CP, and AuCuFe/CP. (c) In 3d spectrum of AuCuIn/CP. (d) Mo 3d spectrum of AuCuMo/CP. (e) Fe 2p spectrum of AuCuFe/CP.

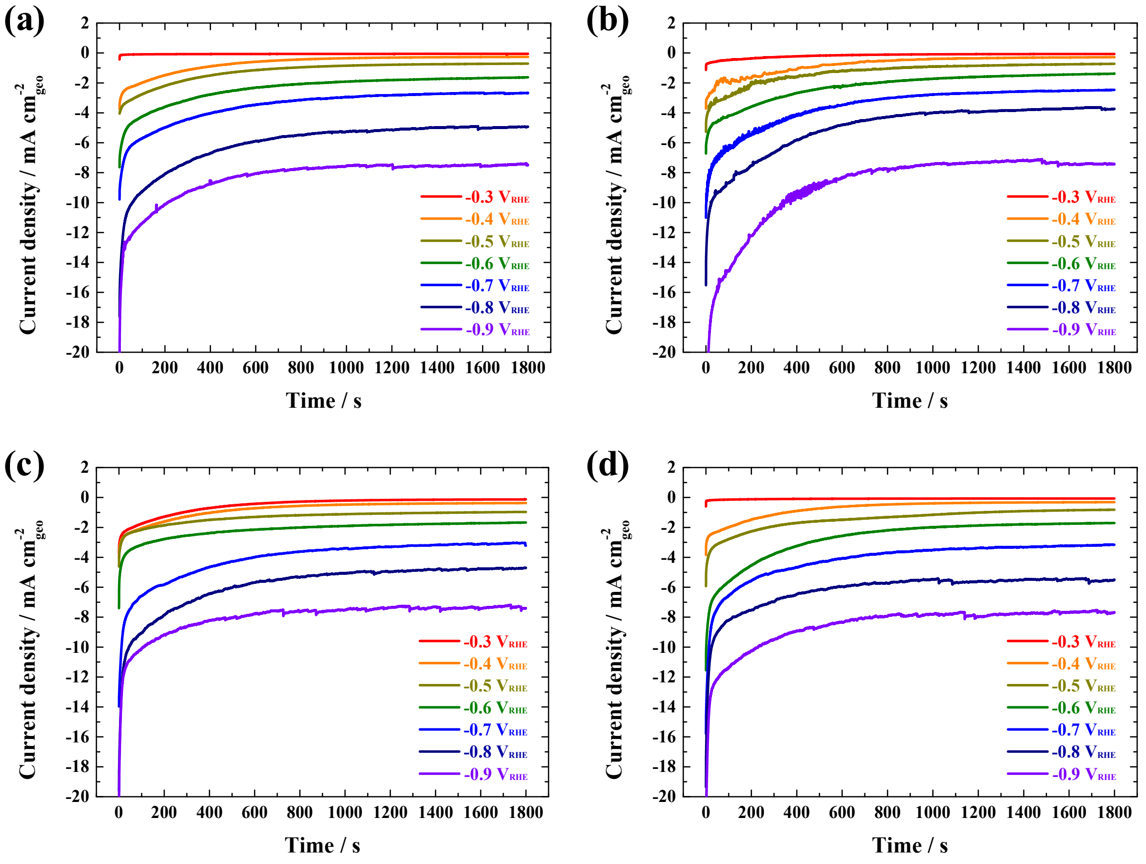

Figure S16. Representative chronoamperometric curves of (a) AuCu/CP, (b) AuCuIn/CP, (c) AuCuMo/CP, and (d) AuCuFe/CP recorded at different applied potentials in CO_2-saturated 0.5 M $KHCO_3$.

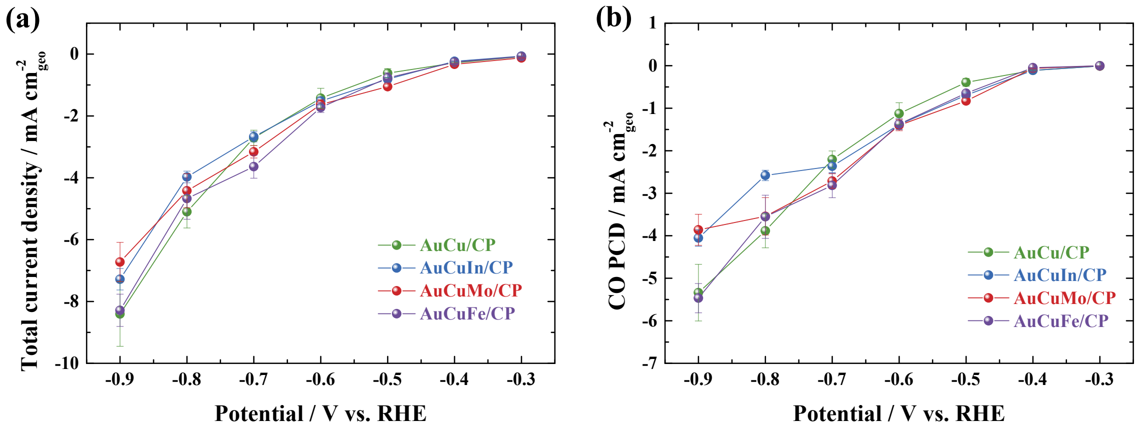

Figure S17. (a) Total current density and (b) CO PCD as functions of applied potential for AuCu/CP, AuCuIn/CP, AuCuMo/CP, and AuCuFe/CP.

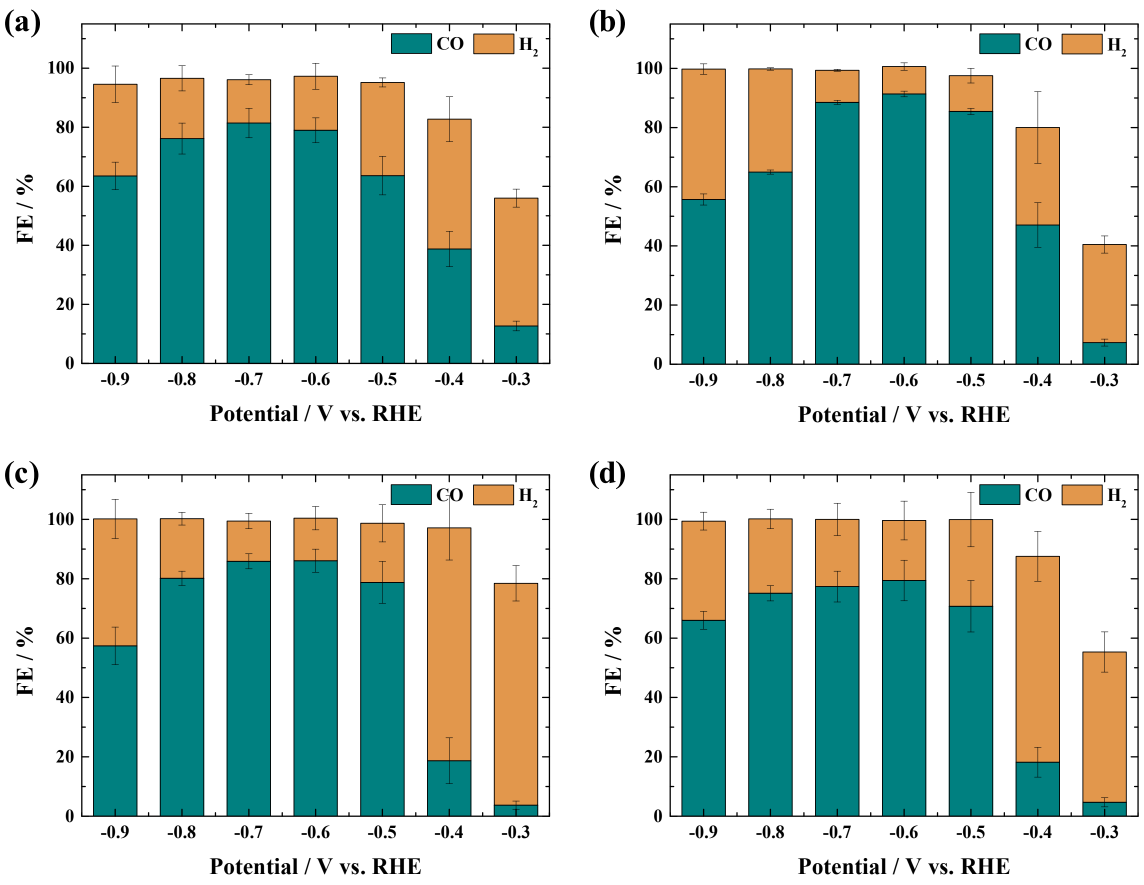

Figure S18. Sum of CO and H_2 FEs as a function of applied potential for (a) AuCu/CP, (b) AuCuIn/CP, (c) AuCuMo/CP, and (d) AuCuFe/CP.

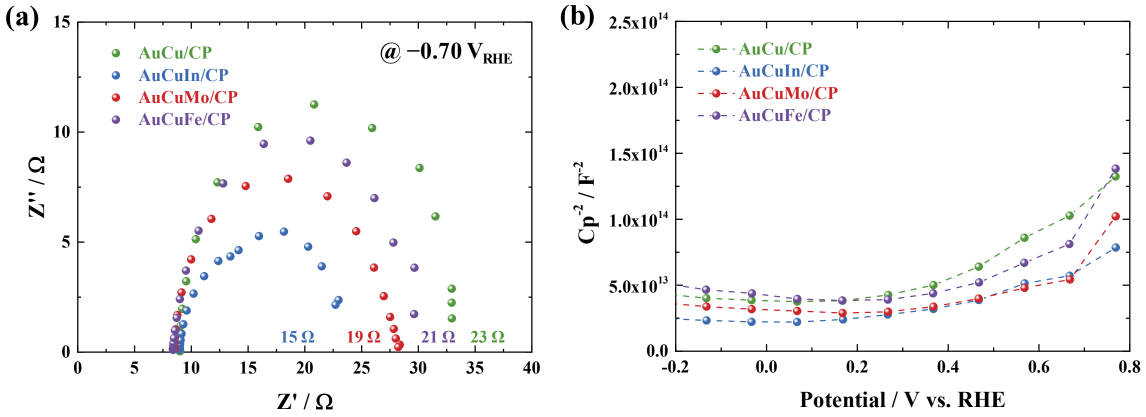

Figure S19. (a) Nyquist plots at −0.70 V_{RHE} and (b) Mott–Schottky plots of AuCu/CP, AuCuIn/CP, AuCuMo/CP, and AuCuFe/CP.

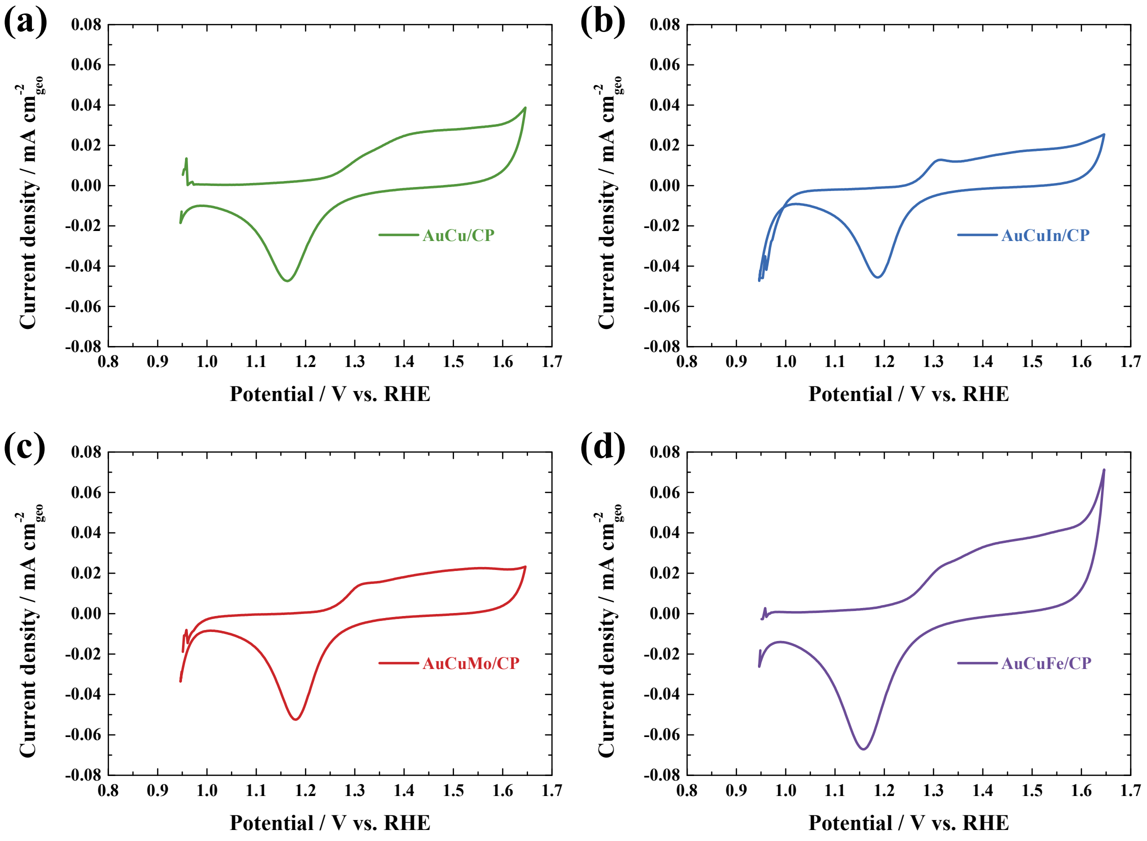

Figure S20. CV curves recorded at 10 mV s^{-1} in N_2-purged 0.1 M NaOH for (a) AuCu/CP, (b) AuCuIn/CP, (c) AuCuMo/CP, and (d) AuCuFe/CP.

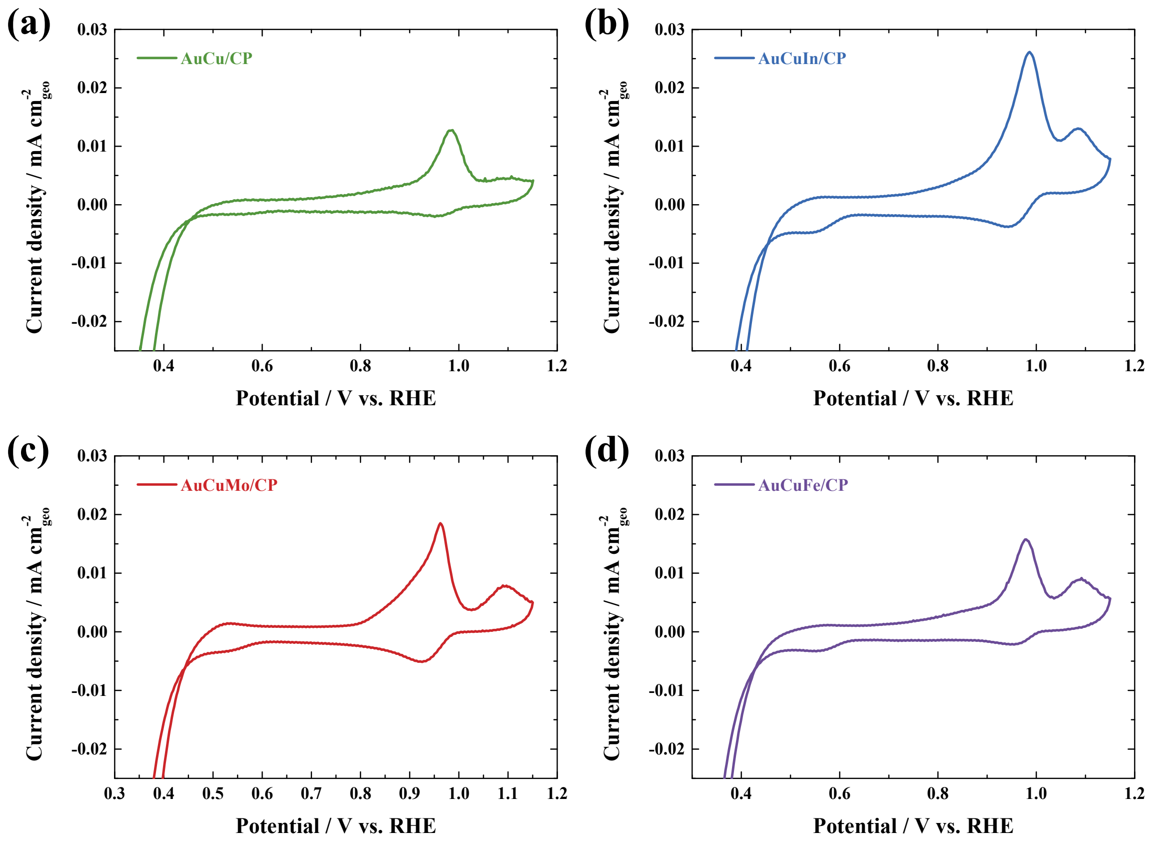

Figure S21. CV curves recorded during the CO stripping test for (a) AuCu/CP, (b) AuCuIn/CP, (c) AuCuMo/CP, and (d) AuCuFe/CP.

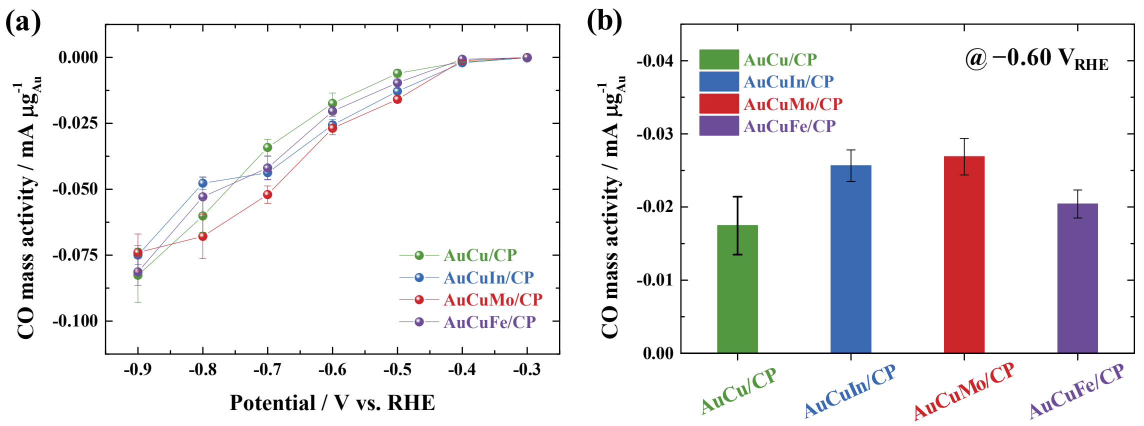

Figure S22. CO mass activity of AuCu/CP, AuCuIn/CP, AuCuMo/CP, and AuCuFe/CP (a) as a function of applied potential and (b) at a fixed potential of −0.60 V_{RHE}.

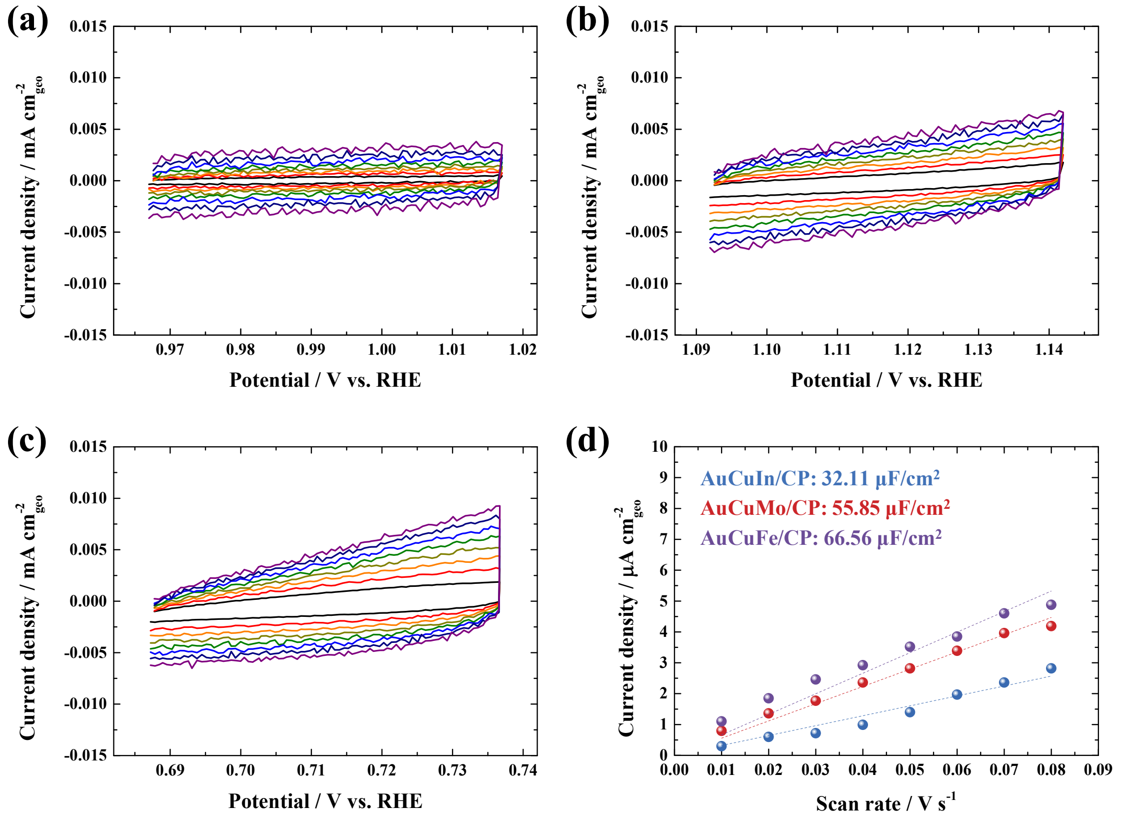

Figure S23. Repeated CV curves recorded at 10–80 mV s^{-1} in N_2-purged 0.5 M $KHCO_3$ for (a) AuCuIn/CP, (b) AuCuMo/CP, and (c) AuCuFe/CP. (d) Results of C_{dl} measurements for AuCuIn/CP, AuCuMo/CP, and AuCuFe/CP.

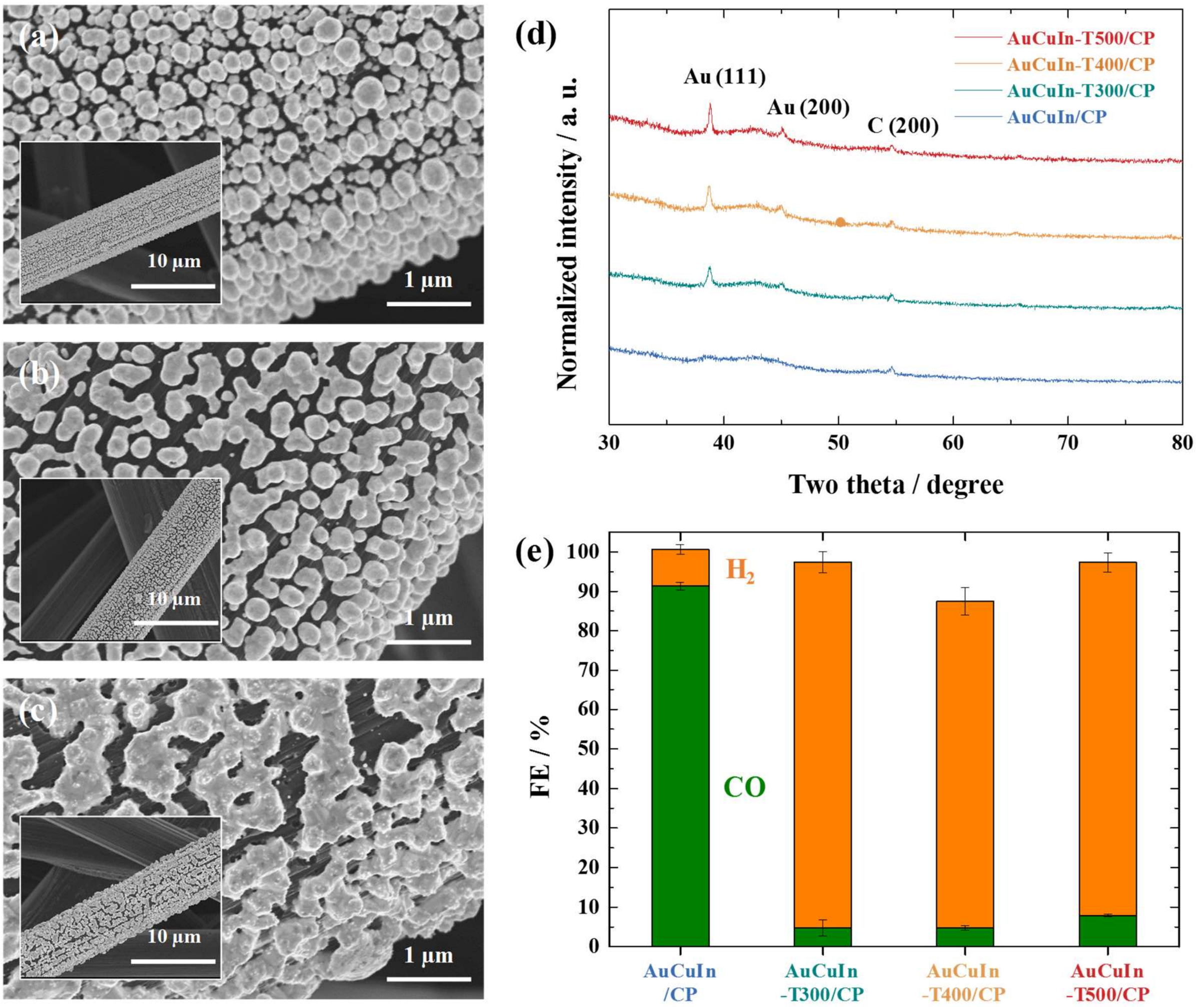

Figure S24. FESEM images of (a) AuCuIn-T300/CP, (b) AuCuIn-T400/CP, and (c) AuCuIn-T500/CP. (d) Normalized-intensity XRD patterns and (e) sum of CO and H_2 FEs as a function of applied potential for AuCuIn/CP and AuCuIn-T#/CP.

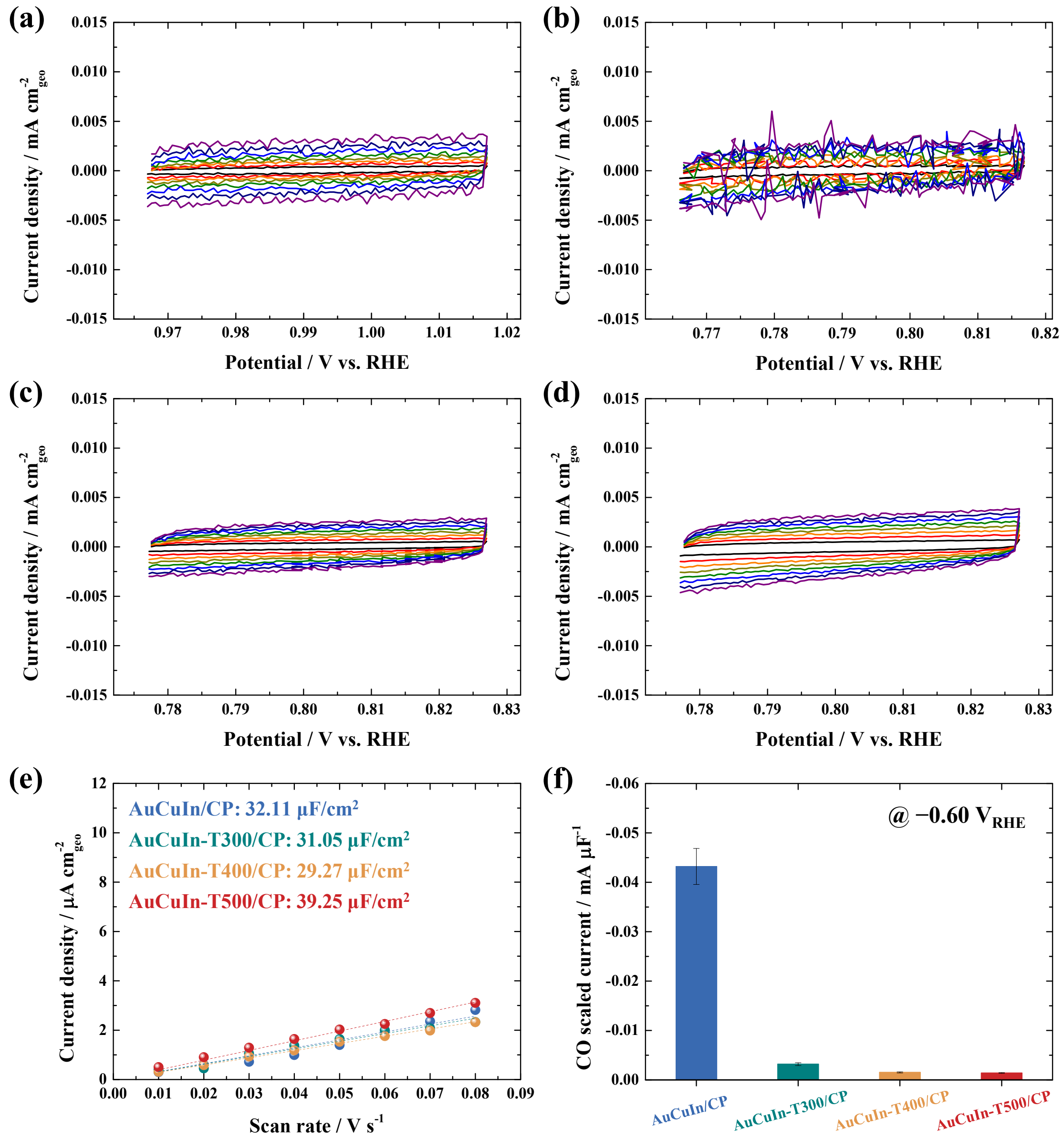

Figure S25. Repeated CV curves recorded at 10–80 mV s^{-1} in N_2-purged 0.5 M $KHCO_3$ for (a) AuCuIn/CP, (b) AuCuIn-T300/CP, (c) AuCuIn-T400/CP, and (d) AuCuIn-T500/CP. (e) Results of C_{dl} measurements and (f) CO scaled current at –0.60 V_{RHE} for AuCuIn/CP and AuCuIn-T#/CP.

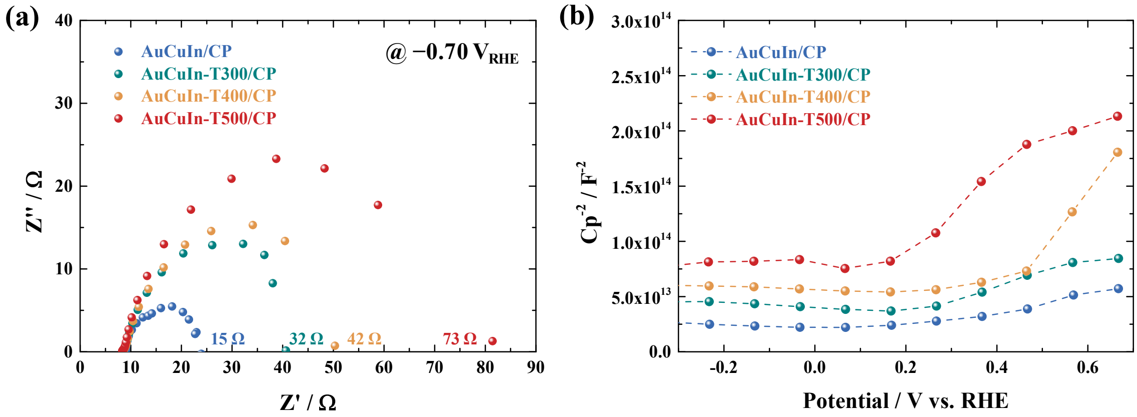

Figure S26. (a) Nyquist plots at –0.70 V_{RHE} and (b) Mott–Schottky plots of AuCuIn/CP and AuCuIn-T#/CP.

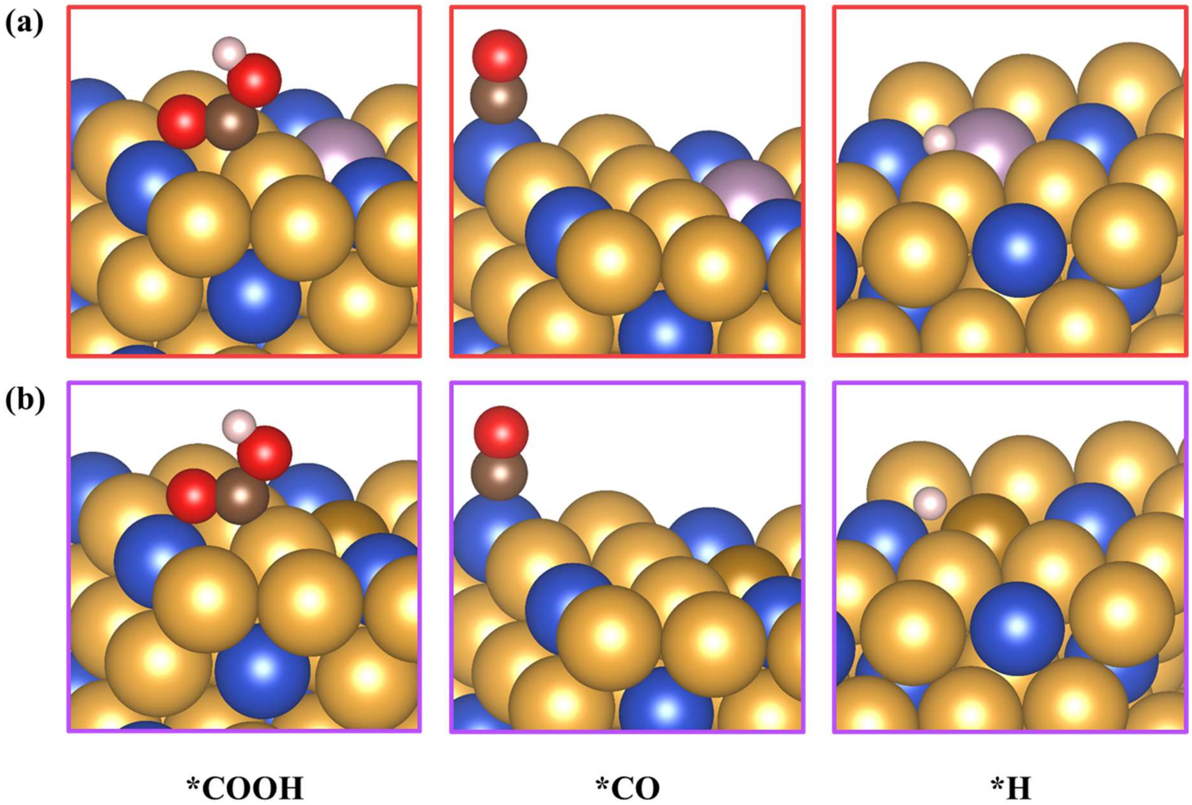

Figure S27. Optimized geometrical structures of *COOH, *CO, and *H on (a) AuCuMo and (b) AuCuFe surfaces.

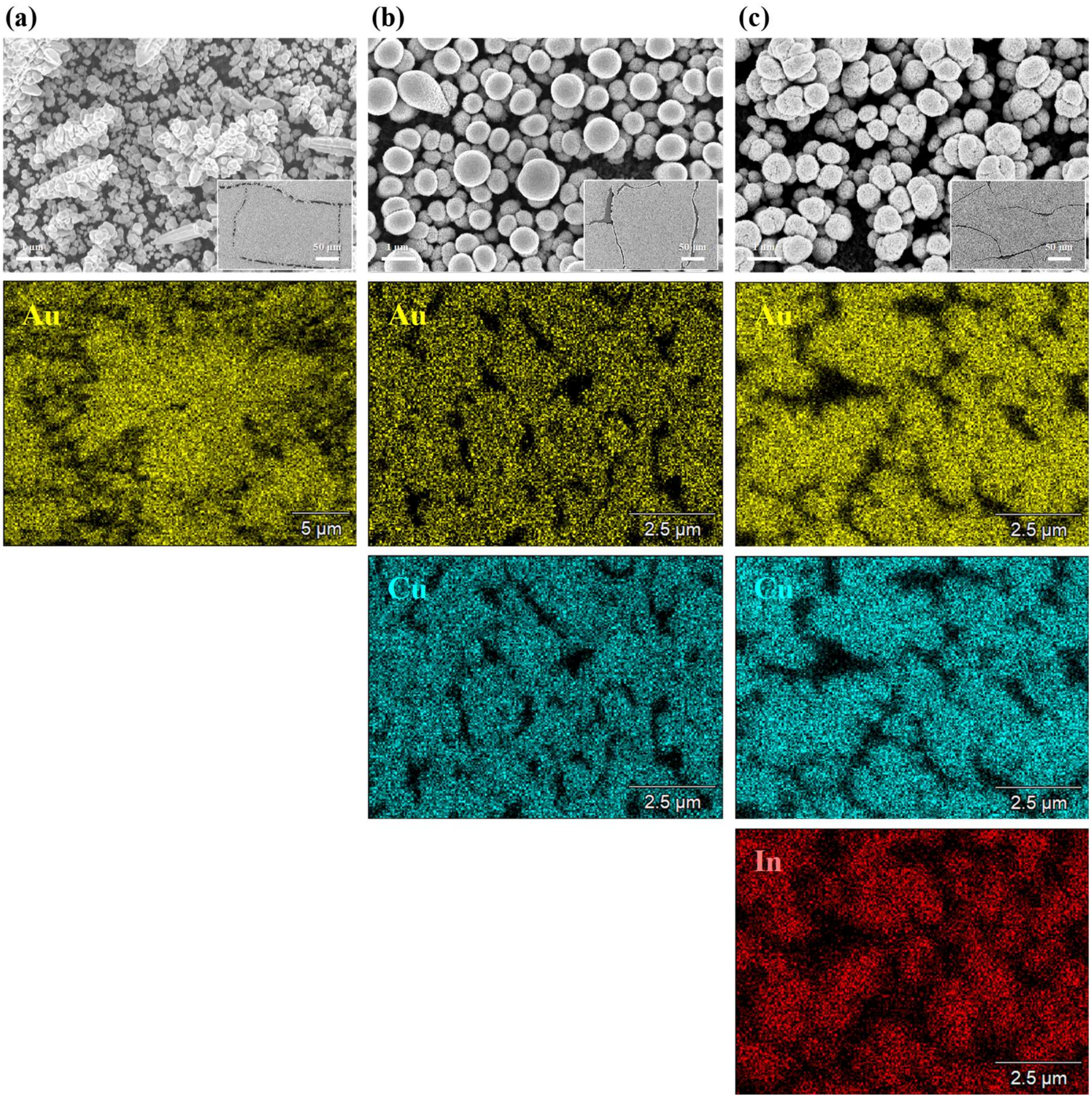

Figure S28. Top-side FESEM images and corresponding elemental mappings of Au, Cu, and In for (a) Au/MPL/CP, (b) AuCu/MPL/CP, and (c) AuCuIn/MPL/CP.

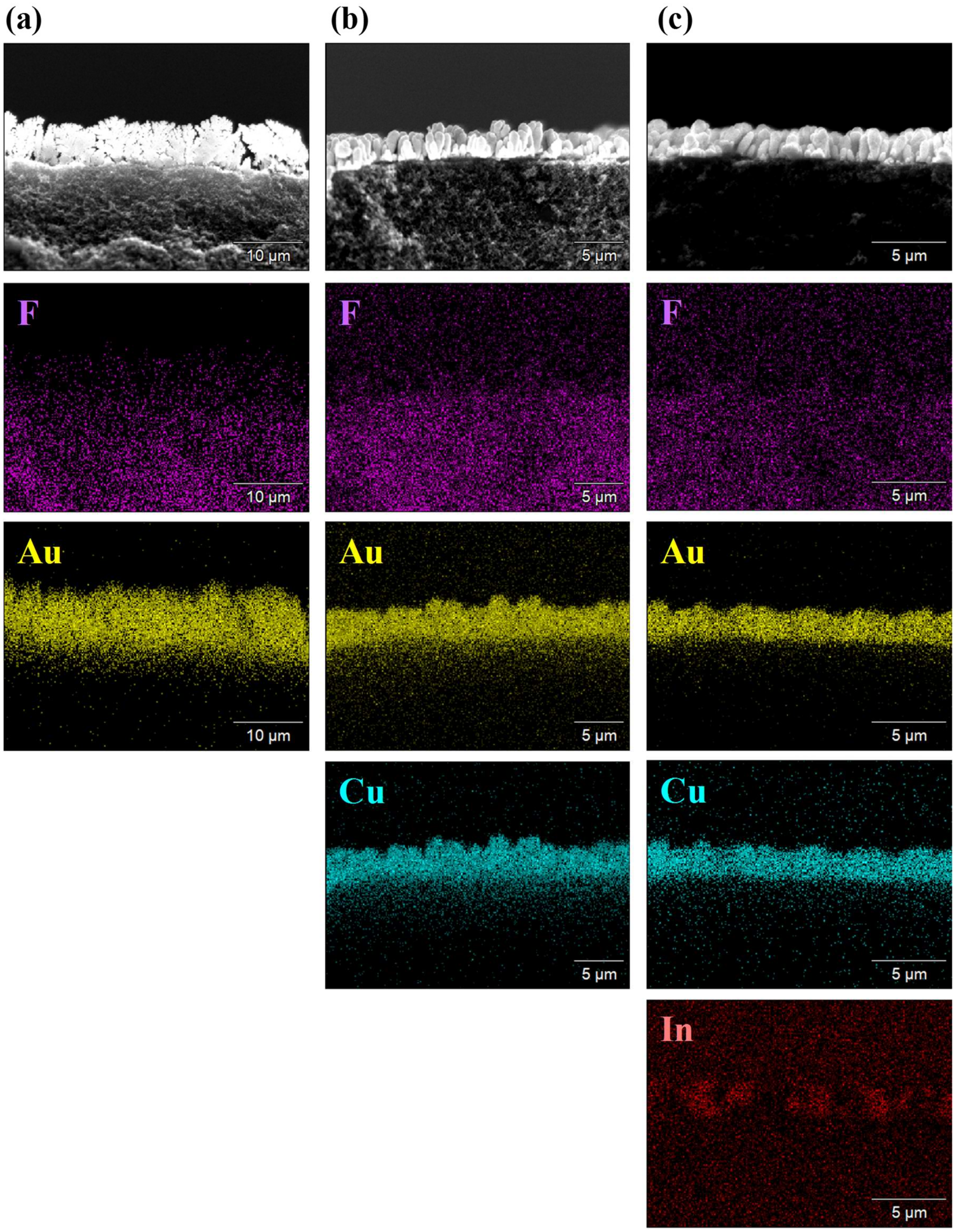

Figure S29. Cross-sectional FESEM images and corresponding elemental mappings of F, Au, Cu, and In for (a) Au/MPL/CP, (b) AuCu/MPL/CP, and (c) AuCuIn/MPL/CP.

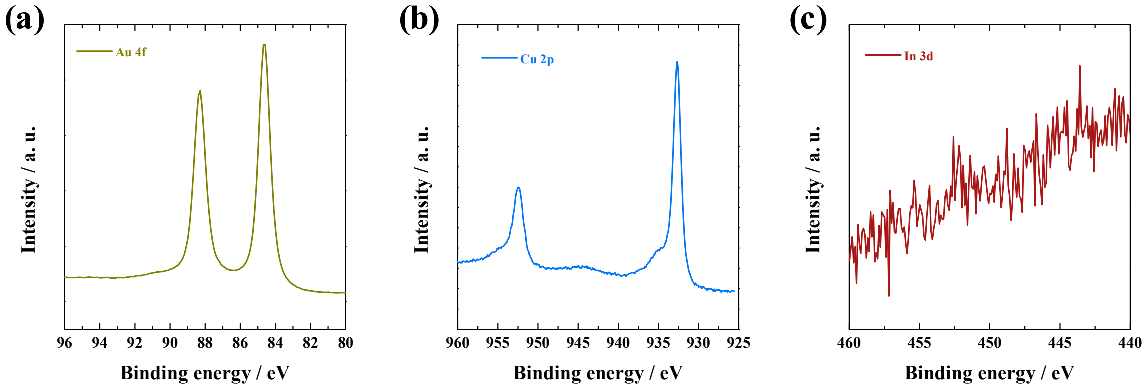

Figure S30. (a) Au 4f, (b) Cu 2p, and (c) In 3d spectra of AuCuIn/MPL/CP.

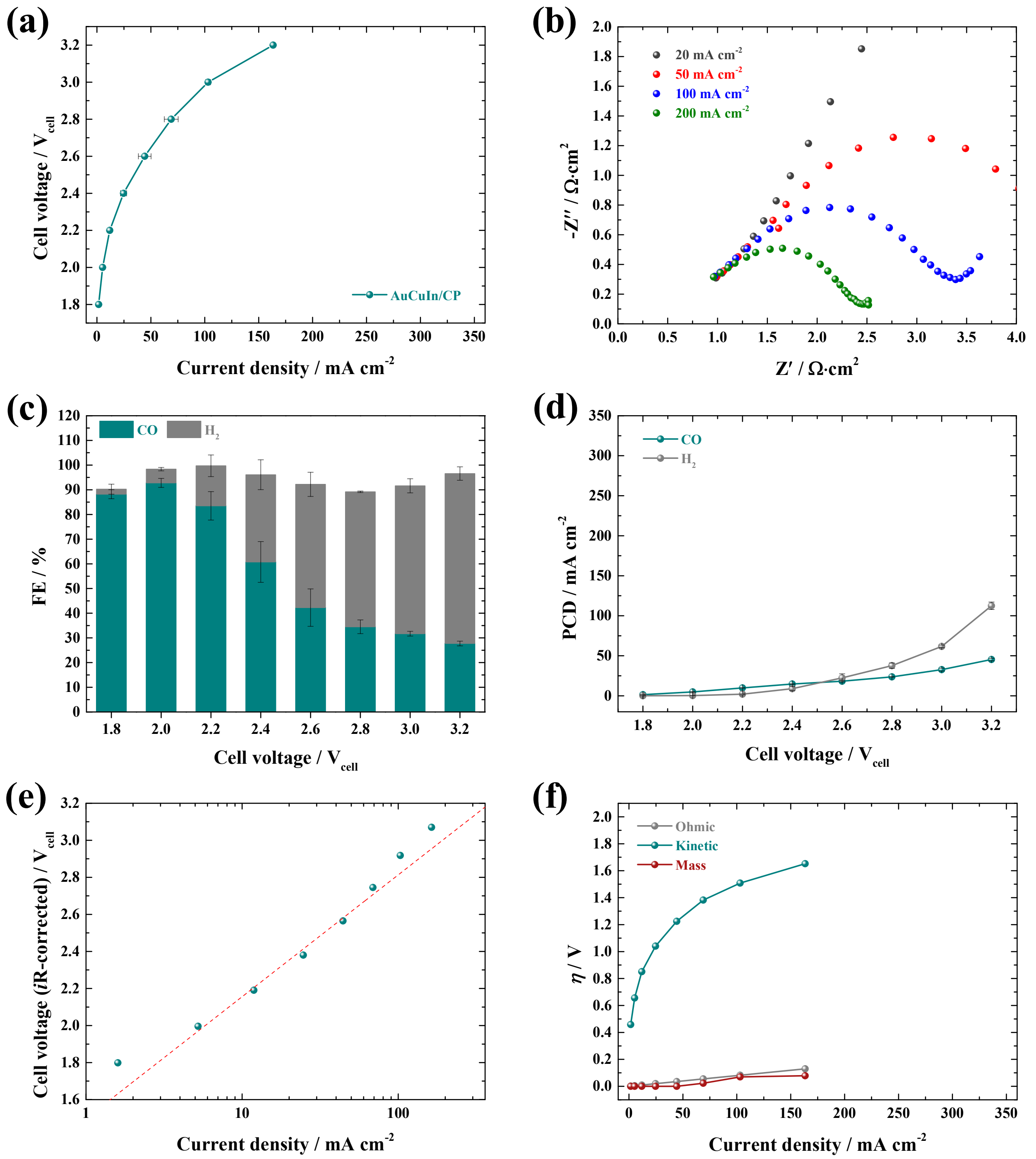

Figure S31. Performance of MEA-based gas-phase CO_2 electrolyzer with AuCuIn/CP cathode. (a) Polarization curve. (b) Nyquist plots. (c) FE and (d) PCD as functions of applied cell voltage. (e) Tafel plot and (f) overpotential subdivision derived from the polarization curve.

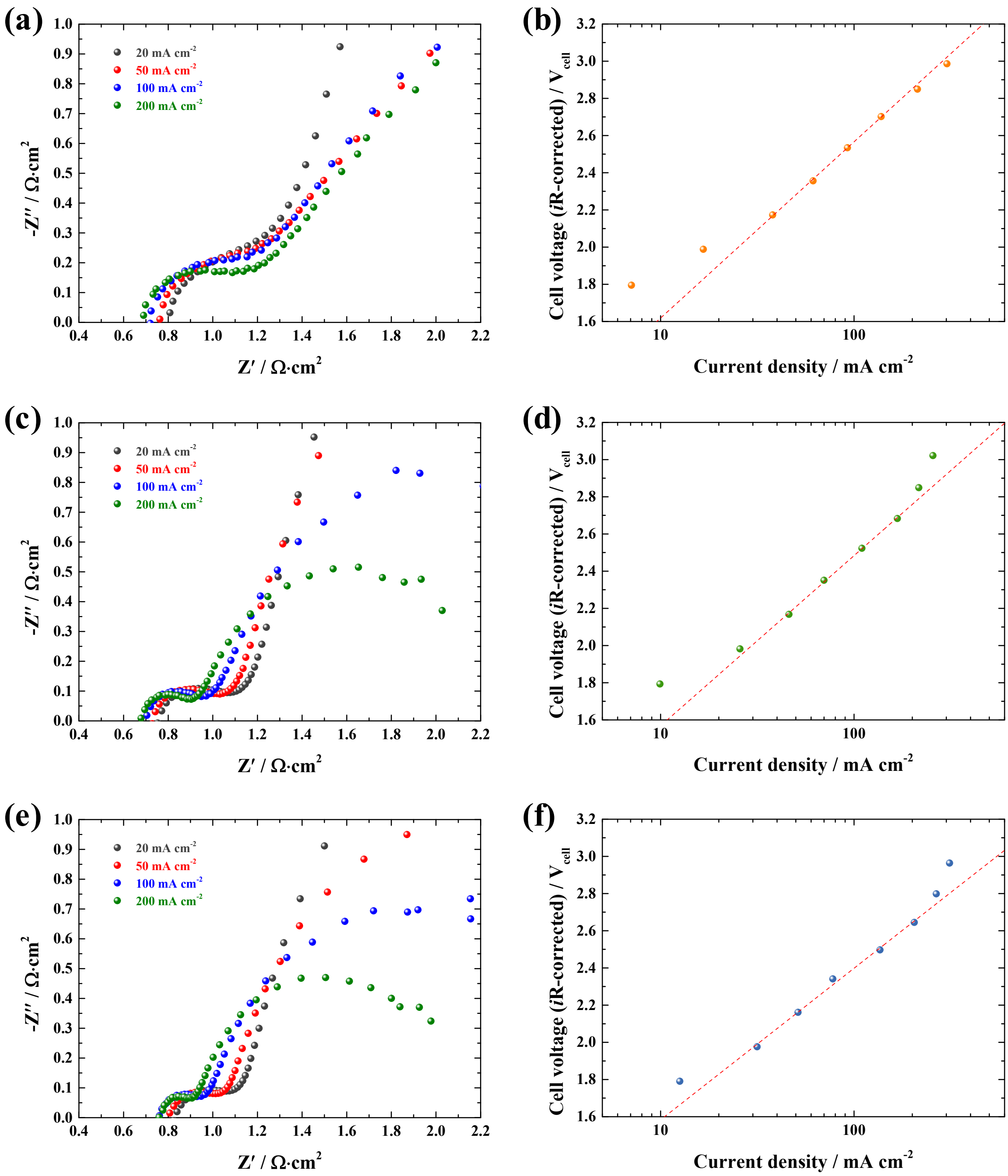

Figure S32. Nyquist (a, c, e) and Tafel (b, d, f) plots of MEA-based CO_2 electrolyzer with (a, b) Au/MPL/CP, (c, d) AuCu/MPL/CP, and (e, f) AuCuIn/MPL/CP cathodes.

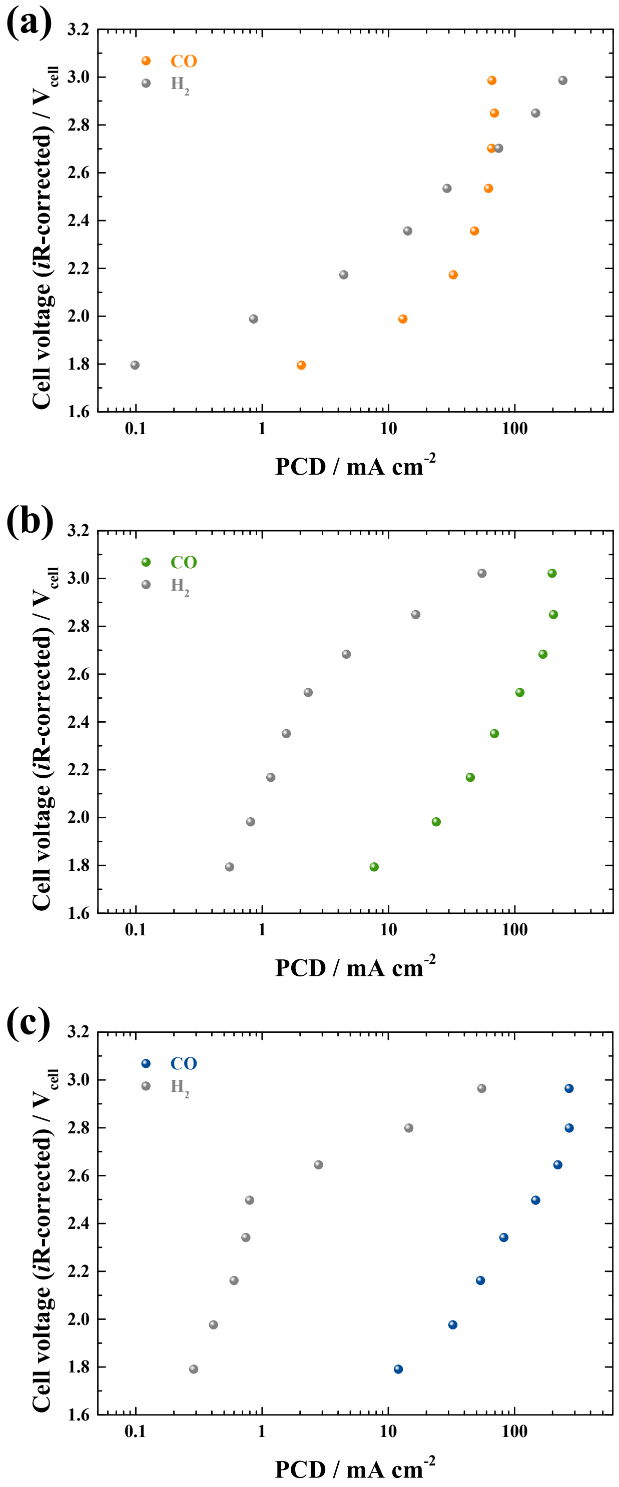

Figure S33. Tafel plots derived from CO and H_2 PCDs for MEA-based CO_2 electrolyzer with (a) Au/MPL/CP, (b) AuCu/MPL/CP, and (c) AuCuIn/MPL/CP cathodes.
